# Local Instrumental Variable Methods to Address Confounding and Heterogeneity when Using Electronic Health Records: An Application to Emergency Surgery

**DOI:** 10.1177/0272989X221100799

**Published:** 2022-05-24

**Authors:** Silvia Moler-Zapata, Richard Grieve, David Lugo-Palacios, A. Hutchings, R. Silverwood, Luke Keele, Tommaso Kircheis, David Cromwell, Neil Smart, Robert Hinchliffe, Stephen O’Neill

**Affiliations:** Department of Health Services Research and Policy, London School of Hygiene & Tropical Medicine, London, UK; Department of Health Services Research and Policy, London School of Hygiene & Tropical Medicine, London, UK; Department of Health Services Research and Policy, London School of Hygiene & Tropical Medicine, London, UK; Department of Health Services Research and Policy, London School of Hygiene & Tropical Medicine, London, UK; University College London, London, UK; University of Pennsylvania, Philadelphia, USA; Department of Health Services Research and Policy, London School of Hygiene & Tropical Medicine, London, UK; Department of Health Services Research and Policy, London School of Hygiene & Tropical Medicine, London, UK; Clinical Effectiveness Unit, Royal College of Surgeons of England, London, UK; College of Medicine and Health, University of Exeter, Exeter, UK; Bristol Surgical Trials Centre, University of Bristol, Bristol, UK; Department of Health Services Research and Policy, London School of Hygiene & Tropical Medicine, London, UK

**Keywords:** cost-effectiveness analysis, emergency surgery, heterogeneous treatment effects, instrumental variable, personalized medicine

## Abstract

**Background:**

Electronic health records (EHRs) offer opportunities for comparative effectiveness research to inform decision making. However, to provide useful evidence, these studies must address confounding and treatment effect heterogeneity according to unmeasured prognostic factors. Local instrumental variable (LIV) methods can help studies address these challenges, but have yet to be applied to EHR data. This article critically examines a LIV approach to evaluate the cost-effectiveness of emergency surgery (ES) for common acute conditions from EHRs.

**Methods:**

This article uses hospital episodes statistics (HES) data for emergency hospital admissions with acute appendicitis, diverticular disease, and abdominal wall hernia to 175 acute hospitals in England from 2010 to 2019. For each emergency admission, the instrumental variable for ES receipt was each hospital’s ES rate in the year preceding the emergency admission. The LIV approach provided individual-level estimates of the incremental quality-adjusted life-years, costs and net monetary benefit of ES, which were aggregated to the overall population and subpopulations of interest, and contrasted with those from traditional IV and risk-adjustment approaches.

**Results:**

The study included 268,144 (appendicitis), 138,869 (diverticular disease), and 106,432 (hernia) patients. The instrument was found to be strong and to minimize covariate imbalance. For diverticular disease, the results differed by method; although the traditional approaches reported that, overall, ES was not cost-effective, the LIV approach reported that ES was cost-effective but with wide statistical uncertainty. For all 3 conditions, the LIV approach found heterogeneity in the cost-effectiveness estimates across population subgroups: in particular, ES was not cost-effective for patients with severe levels of frailty.

**Conclusions:**

EHRs can be combined with LIV methods to provide evidence on the cost-effectiveness of routinely provided interventions, while fully recognizing heterogeneity.

**Highlights:**

- This article addresses the confounding and heterogeneity that arise when assessing the comparative effectiveness from electronic health records (EHR) data, by applying a local instrumental variable (LIV) approach to evaluate the cost-effectiveness of emergency surgery (ES) versus alternative strategies, for patients with common acute conditions (appendicitis, diverticular disease, and abdominal wall hernia).
- The instrumental variable, the hospital’s tendency to operate, was found to be strongly associated with ES receipt and to minimize imbalances in baseline characteristics between the comparison groups.
- The LIV approach found that, for each condition, there was heterogeneity in the estimates of cost-effectiveness according to baseline characteristics.
- The study illustrates how an LIV approach can be applied to EHR data to provide cost-effectiveness estimates that recognize heterogeneity and can be used to inform decision making as well as to generate hypotheses for further research.

## Introduction

Electronic health records (EHRs) offer important opportunities for comparative effectiveness research that can directly inform medical decision making.^1,2^ EHRs offer the possibility of evaluating interventions as provided in practice to all eligible patients. Agencies, such as the National Institute for Health and Care Excellence (NICE), recognize the potential of EHRs,^3^ but to provide useful evidence about comparative effectiveness, two major concerns must be addressed. First, treatment selection according to unmeasured baseline prognostic measures (e.g., disease severity) can make results subject to unmeasured confounding.^4,5^ Second, there may be treatment effect heterogeneity according to patient and contextual characteristics. While approaches for handling heterogeneity according to measured covariates (effect modification) are commonly used, less attention has been given to “essential heterogeneity,” that is, heterogeneous gains according to unmeasured characteristics that influence selection into treatment.^6,7^

The first challenge is unlikely to be addressed by studies that apply traditional risk adjustment methods to provide estimates of comparative effectiveness, as EHRs tend to have inadequate information on case severity.^8,9^ A valid instrumental variable (IV) design can provide accurate estimates of treatment effectiveness, even when there are unmeasured differences between the comparison groups.^10^ If the IV is valid, it encourages receipt of the treatment, but does not have an effect on the outcome, except through treatment receipt. However, a major concern with applying traditional IV approaches, such as 2-stage least squares (2SLS) in the presence of essential heterogeneity, is that the resultant estimates are unlikely to apply to the overall populations or subpopulations of decision-making interest.^10–13^

Local instrumental variable (LIV) approaches can provide estimates of comparative effectiveness that apply to policy-relevant populations.^14–16^ LIV methods can estimate individual-level treatment effects, known as person-centered treatment (PeT) effects, which can then be aggregated over relevant subgroups. LIV methods make the same underlying assumptions as all IV methods but also require that the instrument be continuous.^16^ LIV approaches have been used for comparative effectiveness research as part of bespoke observational studies of educational reforms,^17^ cardiovascular and bariatric surgery,^18,19^ and transfers to intensive care units,^20^ but they have not been applied to EHR data, nor to an economic evaluation. In EHR settings, it is particularly challenging to identify and assess the validity of an IV, given that the data are collected for clinical or administrative rather than research purposes.

These major challenges of using EHRs for comparative effectiveness research are exemplified by the ESORT study,^21^ which aims to evaluate the effectiveness and cost-effectiveness of ES versus nonemergency surgery (NES) strategies, which include antibiotic therapy, nonsurgical procedures (e.g., drainage of abscess), or surgery deferred to the elective (planned) setting. The question as to whether ES or NES strategies are more cost-effective is important, given the high burden of emergency general surgical services and the lack of evidence to inform clinical decision making.^22–24^ Here, an unmet challenge is to identify those patient groups for whom ES is most cost-effective, and conversely those for whom NES alternatives, such as later surgery, may be more worthwhile. Randomized controlled trials (RCTs) have been undertaken for some acute conditions such as acute appendicitis and diverticular disease, but these have included highly selective or small patient samples, whereas for other acute conditions, such as abdominal wall hernia, no RCTs of ES have been conducted.^25–28^

Faced with this evidence gap, the ESORT study uses records from England’s Hospital Episode Statistics (HES) database on emergency admissions to acute National Health Service (NHS) hospitals from 2009 to 2019, for common acute conditions, including the 3 considered in this article, acute appendicitis, diverticular disease, and abdominal wall hernia.^21^ HES for admitted patient care is a database containing administrative, patient, and clinical details of all admissions to hospitals in England’s NHS.^29^ Clinical data on diagnoses and procedures are routinely extracted from discharge summaries for inclusion in local patient information databases, and transferred to HES. The HES database is primarily used for administrative and payment purposes. HES lacks detailed clinical data held locally but has been used widely for research purposes. The ESORT study previously used HES data and found no evidence of differences in the overall clinical effectiveness of ES versus NES strategies.^30^ However, this earlier article did not consider alternative approaches for tackling the confounding that arises with HES data or provide the estimates of relative cost-effectiveness that are essential for decision making.

The aim of this article is to critically examine LIV methods for addressing unmeasured confounding and heterogeneity in evaluating the cost-effectiveness of ES for patients with these 3 conditions from EHR data. The article is structured as follows. First, we provide an overview of the ESORT study. Second, we define the main aspects of the LIV methodology, including application to the ESORT study. Third, we present the results. Fourth, we discuss the key findings, strengths, and limitations of the article and the implications for further research.

## Methods

### Essential Features of the ESORT Study

#### Data sources and study population

The ESORT study uses HES data to evaluate the relative effectiveness and cost-effectiveness of ES versus alternative strategies from the hospital perspective over a 1-y time horizon. The study protocol and statistical analysis plan were developed following the principles of the target trial emulation framework.^21,31^ Briefly, the ESORT study includes patients aged 18 y or older, admitted as an emergency admission via an accident and emergency department, or primary care referral, who were admitted to 175 NHS hospitals in England from April 1, 2010, to December 31, 2019; had the relevant ICD-10 diagnostic codes; and met other inclusion criteria (see Supplementary Table S1).^21,32^

#### Comparator strategies

Admissions were defined as receiving the ES strategy if, according to Office of Population Censuses and Surveys (OPCS) codes, they had a relevant operative procedure within time windows designated by a clinical panel of 3 d (hernia), 7 d (appendicitis), or any time within the emergency admission (diverticular disease).^32^ The NES strategies included medical management, interventional radiology, and operative procedures that did not meet the ES criteria (see Supplementary Table S1).

#### Covariates

Baseline covariates were extracted from HES and included age, sex, ethnicity, the Index of Multiple Deprivation, the Charlson Comorbidity Index,^33^ the secondary care administrative records frailty (SCARF) index,^34^ and teaching hospital status. The SCARF index uses ICD-10 codes to define 32 deficits that cover functional impairment, geriatric syndromes, problems with nutrition, cognition and mood, and medical comorbidities, with severe frailty defined as the presence of 6 or more deficits.^34^ Information was taken from HES data to derive proxy measures of the quality of acute care in each hospital according to rates of 90-d all-cause mortality and emergency readmissions in preceding periods. Subgroups of interest were defined ex ante, drawing on clinical judgment to define those strata anticipated to modify the relative effectiveness and cost-effectiveness of ES. Subgroup definitions were based on the following baseline characteristics: age group, sex, Charlson comorbidity index, SCARF index, diagnostic subcategories, and year of admission.

#### Outcomes

The CEA took an intention-to-treat approach, whereby all patients contributed to the treatment group to which they were assigned at baseline, irrespective of the subsequent treatments received (e.g., planned or unplanned surgery). We reported the mean (95% confidence interval) incremental costs, quality-adjusted life-years (QALYs), and net monetary benefit (INB) at 1 y. Individual-level resource use was extracted from HES data for the index emergency admission and for all subsequent hospital readmissions up to the end of follow-up (death or December 31, 2019). Resource use included the length of the hospital stay, including time in intensive care units, and the use of diagnostic and operative procedures. Resource use items were combined with unit costs (£ GDP, 2019/20) to calculate total costs per patient (see section 1 and Tables S2, S3, and S4 in the supplementary materials). All unit costs were inflated to 2019–20 prices (£ GBP) using UK’s GDP deflator published by HM Treasury.^35^

Survival time up to 1 y was calculated for all patients from HES records linked to the Office for National Statistics death data. Health-related quality of life (HRQoL) data were not available from HES, and so QALYs were calculated by combining the survival time with HRQoL estimates from the literature (see sections 2 and 3 and Tables S5 and S6 in the supplementary materials). We derived each patient’s QALYs at 1 y using the area under the curve approach,^36^ which allowed HRQoL to decrease to baseline levels following an emergency readmission, but assumed that HRQoL levels recovered following hospital discharge. HRQoL levels were adjusted to reflect the patient’s age and gender, and were assumed to be zero for patients who died over the follow-up period.^37,38^ The study’s cost-effectiveness metric was the INB of ES versus NES, calculated by multiplying the incremental QALYs by a NICE recommended willingness-to-pay threshold of £20,000 per QALY and subtracting from this the incremental cost.^3^

We now present the main elements of the LIV design (in the following section). We then discuss how PeT effects, average treatment effect (ATE), and conditional ATEs (CATEs) were estimated using LIV and contrast the results against 2 alternative methods for estimating the ATE—2-stage residual inclusion and GLM regression—which make different assumptions about confounding and heterogeneity.

### IV Estimation

#### Overview

A valid instrument must be associated with treatment assignment (relevance condition) (i), the IV must be independent of unmeasured confounders (exchangeability condition) (ii), the IV must influence the outcomes only through treatment assignment (exclusion-restriction assumption) (iii), and the IV must have the same direction of effect on the probability of which treatment is received, irrespective of the level of the IV (monotonicity) (iv).^10,12^ The most widely used IV approach, 2SLS, estimates the average treatment effect (ATE) when effects are homogeneous. If there are heterogeneous treatment effects, and the IV is binary, 2SLS reports a local ATE (LATE) or a weighted average of LATEs with a continuous IV,^39,40^ requiring careful interpretation of the estimated effects in light of the LATE estimand.

### Two-Stage Residual Inclusion

2-stage residual inclusion (2SRI) is an IV approach that relies on concepts that support control function methods in an attempt to control for unmeasured confounding.^41^ This approach uses residuals from a first-stage regression for treatment assignment, in a second-stage outcome model.^41^ Unlike 2SLS, the 2SRI approach, when applied to a binary treatment, aims to estimate the ATE rather than LATEs. However, concerns have been raised that this approach may provide biased estimates of the ATE due to the necessity to extrapolate the residuals when constructing counterfactuals, and that it is sensitive to misspecification of the functional form underlying the residuals.^42^ Here, we address the latter concern by using generalized residuals, which have been shown to minimize the bias in estimating the ATE.^42^ Nonetheless, although 2SRI can, in some circumstances, provide accurate estimates of the ATE, it is not specifically recommended for exploring heterogeneity.^41^

#### Estimating person-level effects using LIV methods

We also consider an LIV method that can estimate ATEs, subgroup effects, and personalized treatment effects, in the presence of unmeasured confounding and heterogeneity, and can extend to nonlinear outcomes such as costs and QALYs.^6,43^

Heckman and Vytlacil^14–16^ showed that LIV methods can identify effects for “marginal” patients, those who are in equipoise with respect to the treatment assignment decision, provided a valid, continuous instrument is available. These individuals’ propensity for treatment (PS), based on the levels of their observed covariates and IV, just balance with a normalized version of the unmeasured confounders (*V*) discouraging treatment, such that a small (marginal) change in the IV is sufficient to nudge them into the treatment group (where D = 1 [i.e., ES] if PS > V and 0 [NES] otherwise). Contrasting outcomes for individuals with marginally different values of the IV, but who are otherwise identical in measured and unmeasured covariates at different levels of the IV, identifies a series of marginal treatment effects (MTEs). The MTE is equivalent to the conditional LATE for infinitesimally small changes in the normalized unobserved confounder, *V.*^44^ MTEs can then be aggregated to obtain the ATE and CATEs for subgroups.^16^

The LIV method relies on correctly modeling the relationships of the covariates and the IV with both the treatment and the outcome, typically using parametric models.^45,46^ If the treatment assignment model is misspecified, the second-stage model will use biased estimates of the PS, thus introducing bias into the subsequent effect estimates. Similarly, if the outcome model is misspecified, the estimated MTEs may not represent the true MTEs, as they will have been derived as the derivative of an incorrect outcome model $MTE=\frac{\partial E(Y|X=x,Z=z)}{\partial ps}$ While the “true” model specifications are unknown, considering alternative specifications, visually inspecting the models’ predictions versus actual values, and considering the root mean squared error (RMSE) of the predictions, in addition to using standard model diagnostic approaches such as Hosmer-Lemeshow^47^ and Pregibon^48^ tests for generalized linear models (GLMs), can be helpful in minimizing the risk of misspecification.

Basu^43,49^ extended the LIV approach by using the individual patient’s observed treatment status to obtain personalized effect estimates. The key insight underlying this approach is that for each individual patient, some levels of the normalized unobserved confounder would be inconsistent with the observed treatment decision for that individual, given their observed characteristics and the level of the IV.^43^ For instance, if an individual with high propensity for ES according to observables (e.g., age) were observed to receive NES, it is reasonable to assume that the discouragement according to unobserved confounders must have exceeded the propensity for ES (i.e., PS < V if D = 0). MTEs that imply a lower level of unobserved confounding can thus be ruled out, narrowing the set of MTEs that could plausibly represent the individual’s effect. The PeT effect for an individual is obtained by aggregating the remaining MTEs. PeT effects therefore account for individual treatment choices and the circumstances under which individuals are making those choices. The PeT effects can then be aggregated to obtain higher-level estimands (e.g., ATE and CATEs).^43,49^ (For full details and implementation in this study, see section 4 of the supplementary materials).

#### Developing IV and LIV approaches within the ESORT study

The ESORT study adopted an IV approach to evaluate ES from US claims data,^50^ following pharmacoepidemiological research in taking clinician preference as an instrument for treatment receipt.^51,52^ In the ESORT study, the IV was the hospital’s tendency to operate (TTO), which reflects practice variation across hospitals in ES rates for these conditions (see Supplementary Figure S1). For each qualifying emergency admission, the TTO was defined as the proportion of eligible emergency admissions in that specific hospital who received ES in the previous 12 mo, thus requiring that the hospital’s past preference for ES strongly predicts treatment choice for the current patient. The rationale for the IV design is that, after adjustment for observed characteristics, the patients’ baseline prognosis is similar across hospitals with different TTO levels.^51^ Hence, the patients can be “randomized” between the ES and NES strategies according to the hospital’s TTO.

**Figure 1:**
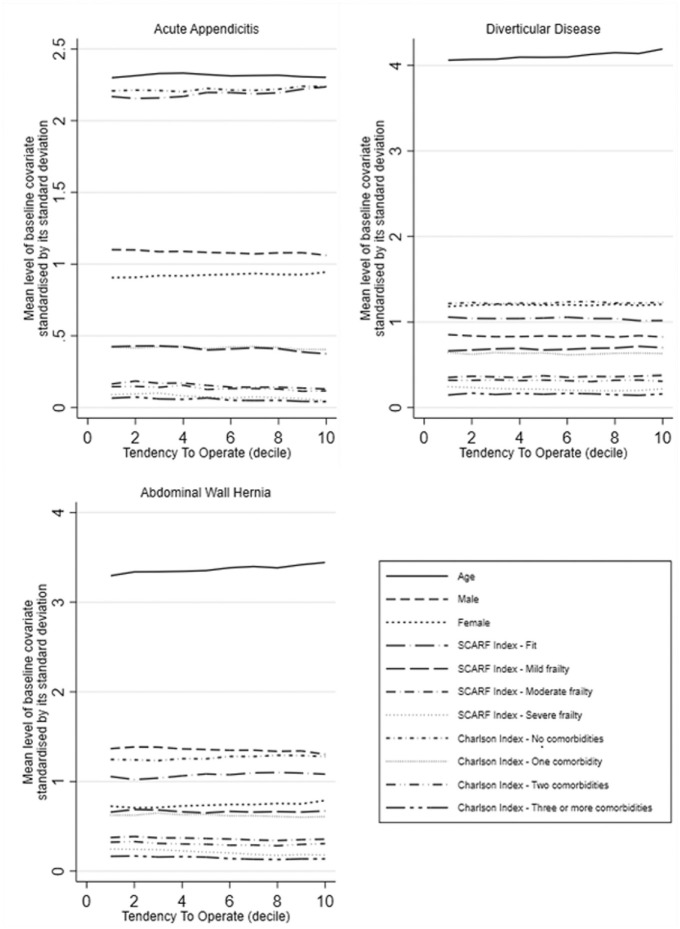
Mean level of rescaled baseline covariates according to the level of the instrumental variable.

While Keele et al.^50^ validated this IV within US claims data, we carefully considered whether each of the above underlying assumptions were met within the EHR data for the ESORT study. We assessed the relevance of the hospital’s TTO with a weak instrument test that is robust to heteroscedasticity and clustering (see Supplementary Table S7 in Supplemental materials).^53^ Assumptions (ii), (iii), and (iv) are untestable. The IV would fail the exclusion-restriction condition (assumption iii) if patients admitted to hospitals with high TTO received better care (e.g., postoperative care) leading to lower mortality or shorter stays (and hence costs), regardless of the treatment received, which seems unlikely. However, to increase the plausibility of assumptions (ii) and (iii), we adjusted for a rich set of potential confounders, including proxies for the quality of acute care in each hospital (see section S5 in the supplementary materials). We assessed the extent to which observed prognostic covariates differed across levels of the instrument (see Figure 1). Imbalances observed in measured covariates across levels of the TTO would raise concerns about assumptions (ii) and (iii). We also observed a strong positive, linear relationship between the hospital-level TTO and receipt of ES for all 3 conditions, providing support for assumption (iv).

### Statistical and Sensitivity Analyses

The LIV estimated the PeT effects of ES versus NES on costs and QALYs for each individual, allowing for treatment effect heterogeneity and confounding.^27–30^ These were aggregated to report the effects of ES overall and for each prespecified subgroup of interest. Probit regression models were used to estimate the initial propensity score (first stage), whereas GLMs were applied to the cost and QALY data, with the most appropriate chosen according to RMSE (see Supplementary Table S7). Hosmer-Lemeshow and Pregibon tests were also used to check the model fit and appropriateness.^47,48^ For the QALY endpoint, the logit link and binomial family were selected (all 3 conditions) and, for costs, the log link and Gaussian family (appendicitis and diverticular disease) and the identity link and gaussian family (hernia). Models at both stages adjusted for the above baseline measures, time period, and proxies for hospital quality, defined by rates of emergency readmission and mortality in 2009 to 2010 (time constant) and in the year prior to the specific admission concerned (time-varying; see section 5 of the supplementary materials).

Overall estimates of incremental costs, QALYs, and INB were reported with standard errors and confidence intervals (CIs) obtained with the nonparametric bootstrap (300 replications), allowing for the clustering of individuals within hospitals and the correlation of individual-level costs and effects. The individual-level estimates of incremental costs and QALYs were also plotted on the cost-effectiveness plane, stratified by subgroups of policy relevance.

The 2SRI and risk-adjustment (GLM regression) approaches took the same approach to model specification and selection (including covariates used for confounding adjustment) to report overall estimates of incremental costs and QALYs and INB. The proportion of missing data across the 3 cohorts was low, with less than 5% missing values for all baseline covariates, other than ethnicity (10% in the appendicitis cohort); thus, a complete case analysis was performed.^54^

#### Sensitivity analyses

Sensitivity analyses were undertaken to assess whether the results from the main analysis were robust to alternative definitions and assumptions. First, the study adjusted for “quality of care” using external hospital performance measures from the National Emergency Laparotomy Audit (NELA).^55–57^ Second, we considered the sensitivity of our findings to the potential for under- or overestimating costs from EHR data by increasing all costs by 10% (SA2) and to reducing them by 10% (SA3). Third, we considered an alternative approach to QALY calculation that used linear interpolation between the baseline admission, and 1-y follow-up (SA4). Fourth, we considered a longer time horizon of 5 y, by restricting the sample to those patients who were admitted from 2010 to 2014 (SA5).

#### Ethics approval

The research was approved by the London School of Hygiene and Tropical Medicine ethics committee (ethics reference No. 21687). The study involved the secondary analyses of existing pseudo-anonymized data and did not require UK National Ethics Committee approval.

## Results

The study included 268,144 (appendicitis), 138,869 (diverticular disease), and 106,432 (hernia) patients. The proportions of patients who had ES were 92.3% (appendicitis), 11.4% (diverticular disease), and 58.8% (hernia). The patients with acute appendicitis who had ES were on average younger and more likely to be fit and without comorbidities as compared with those who had NES strategies. For patients with diverticular disease, patients who had ES were less likely to be fit but were of similar age and comorbidity profile to those in the NES groups. For patients with hernia, a higher proportion of women had ES. Other baseline characteristics were similar between the comparison groups (Table 1).

**Table 1:** Baseline Characteristics of Patients in the Cohorts

	Acute Appendicitis (*n* = 268,144)		Diverticular Disease (*n* = 138,869)		Abdominal Wall Hernia (*n* = 106,432)	
	ES (*n* = 247,506)	NES (*n* = 20,638)	ES (*n* = 15,772)	NES (*n* = 123,097)	ES (*n* = 62,559)	NES (*n* = 43,873)
Gender, *n* (%)						
Male	134,270 (54)	10,409 (50)	7,074 (45)	49,922 (41)	37,522 (60)	31,341 (71)
Female	113,224 (46)	10,228 (50)	8698 (55)	73,172 (59)	25,035 (40)	12,530 (29)
Age, y, mean	38	47	64	64	63	62
SCARF index, *n* (%)						
Fit	206,796 (84)	15,015 (73)	6197 (39)	65,911 (54)	33,014 (53)	23,871 (54)
Mild frailty	34,544 (14)	4052 (20)	5631 (36)	38,851 (32)	19,608 (31)	13,104 (29)
Moderate frailty	5041 (2)	1155 (6)	2706 (17)	13,433 (11)	7360 (12)	4987 (11)
Severe frailty	1125 (0)	416 (2)	1238 (8)	4902 (4)	2577 (4)	1911 (4)
Charlson index, *n* (%)						
0 comorbidities	207,525 (84)	15,321 (74)	9789 (62)	73,457 (60)	39,216 (63)	26,297 (60)
1	35,721 (14)	3989 (19)	4482 (28)	35,106 (29)	17,494 (28)	12,163 (28)
2	3715 (2)	1035 (5)	1222 (8)	11,454 (9)	4792 (8)	4169 (10)
3+ comorbidities	545 (0)	293 (1)	279 (2)	3080 (3)	1057 (2)	1244 (3)

ES, emergency surgery; NES, nonemergency surgery; SCARF, secondary care administrative records frailty.

The most prevalent forms of ES are listed in Supplementary Table S8. Most patients in the NES strategy groups did not have an operative procedure.

Table 2 presents the unadjusted costs of ES and NES. For patients with diverticular disease, the average total costs for the ES group at 1 y were higher than for the NES group (£16,498 v. £4673), reflecting the higher initial admission costs, including operative costs. For the other 2 conditions, the average 1-y costs of ES versus NES were similar, with the higher operative costs of ES offset by higher readmission costs following the NES strategy (see Supplementary Table S8). For patients with diverticular disease, before any case-mix adjustment, the proportion of patients who had died by 1 y was higher in the ES versus NES group (see Supplementary Figure S2).

**Table 2:** Unadjusted Costs of ES and NES Strategies (£GBP 2019–2020)

	Acute Appendicitis (*n* = 268,144)		Diverticular Disease (*n* = 138,869)		Abdominal Wall Hernia (*n* = 106,432)	
	ES (*n* = 247,506)	NES (*n* = 20,638)	ES (*n* = 15,772)	NES (*n* = 123,097)	ES (*n* = 62,559)	NES (*n* = 43,873)
Index admission						
Bed-day costs (£), mean (*s*)	1613 (2080)	1850 (3147)	10,637 (12,919)	1880 (2511)	2249 (7036)	1181 (3853)
Cost of diagnostic procedures (£), mean (*s*)	28.0 (54.2)	57.8 (69.1)	108 (104)	86.5 (81.4)	20.3 (52.3)	18.2 (45.1)
Cost of operative procedures (£), mean (*s*)	1132 (127)	192 (429)	1947 (938)	1.68 (32.8)	809 (244)	42.3 (209)
Total costs in index admission (£), mean (*s*)	2774 (1974)	2101 (3213)	12,690 (13,124)	1967 (2537)	3079 (7066)	1242 (3938)
Readmissions up to 1 y						
Patients with 1+ readmissions, *n* (%)	66,446 (26.8)	10,895 (53.0)	10,100 (64.2)	90,300 (74.4)	25,947 (41.5)	31,997 (72.9)
Bed-day costs (£), mean (*s*)	541 (2594)	1408 (4208)	3444 (8028)	2422 (6167)	1786 (5998)	2581 (7413)
Cost of diagnostic procedures (£), mean (*s*)	22.5 (80.2)	70.2 (142)	94.4 (149)	146 (174)	33.5 (100)	45.7 (120)
Cost of operative procedures (£), mean (*s*)	18.5 (139)	178 (419)	270 (628)	137 (496)	62.7 (242)	406 (457)
Total costs in readmissions, mean (*s*)	582 (2650)	1656 (4338)	3808 (6374)	2706 (6743)	1882 (6061)	3033 (7468)
Total costs at 1 y, mean (*s*)	3355 (3519)	3757 (5658)	16,498 (16,027)	4673 (7145)	4961 (9666)	4275 (8680)

ES, emergency surgery; NES, nonemergency surgery.

### IV Diagnostics

The hospital’s TTO was strongly correlated with ES receipt for all 3 conditions, after case-mix adjustment (see Table 3). For the 3 conditions, the F statistic ranged from 135 (appendicitis) to 735 (hernia) versus the commonly applied threshold of 10.^58^ Thus, the hospital’s past preference for ES strongly predicts treatment choice for the current patient. The mean levels of the baseline covariates (rescaled) were similar across the TTO levels (see Figure 1), which makes it more plausible that the IV also balances unobserved covariates.

**Table 3:** Instrumental Variable Strength for the Hospital-Level Tendency-to-Operate within the HES Data (2009–2019) for Emergency Admissions That Met the ESORT Study Inclusion Criteria for Each of the 3 Conditions

Condition	Montiel-Pflueger Robust Weak Instrument Test F-Statistic
Appendicitis	135
Diverticular disease	206
Abdominal wall hernia	735

### Overall Cost-Effectiveness Results by Method

Table 4 reports the estimated incremental costs and QALYs and the INB according to the intention-to-treat principle for the overall population using regression adjustment, 2SRI, and the LIV approach. For patients with appendicitis and hernia, all 3 methods reported mean INBs close to zero. For patients with diverticular disease, the results differed by method. The regression adjustment and the 2SRI approaches reported that ES has positive incremental costs, negative incremental QALYs, and negative INBs with 95% CIs below zero (Table 4). By contrast, the LIV results show that there was considerable uncertainty in the overall cost-effectiveness estimates for all 3 conditions, with 95% CIs around the INBs that included zero (Table 4). For acute appendicitis, the incremental QALYs and costs were also close to zero (Table 4). For patients with diverticular disease, the LIV approach reported that, on average, ES led to a cost reduction (−£1724), QALY gain (0.047), and a positive INB (£2664). For patients with abdominal wall hernia, the LIV approach reported that the positive incremental costs of ES (£891) were offset by moderate QALY gains (0.0386; see Supplementary Figure S3).

**Table 4:** 

	Acute Appendicitis (*n* = 268,144)	Diverticular Disease (*n* = 138,869)	Abdominal Wall Hernia (*n* = 106,432)
INB			
Unadjusted differences	1431 (1259, 1603)	−13,088 (−13,509, −12,668)	−303 (−469, −137)
GLM	−165 (−287, −42)	−12,381 (−12,848, −12,058)	−50.1 (−241, 141)
GLM-2SRI	281 (−743, 1306)	−7496 (−12,230, −2763)	−1474 (−3038, 2995)
LIV	−86.2 (−1163, 991)	2664 (−4298, 9626)	−119 (−1282, 1043)
Incremental costs			
Unadjusted differences	−413 (−513, −312)	11,857 (11,486, 12,228)	674 (548, 800)
GLM	318 (213, 424)	11,266 (10,905, 11,626)	483 (318, 649)
GLM-2SRI	762 (−73.5, 1598)	5990 (1371, 10,609)	1645 (295, 2995)
LIV	−109 (−1130, 913)	−1724 (−7878, 4430)	891 (20.7, 1,762)
Incremental QALYs			
Unadjusted differences	0.0509 (0.0462, 0.0556)	−0.0616 (−0.0672, −0.0559)	0.0186 (0.0150, 0.0221)
GLM	0.00767 (0.00550, 0.00983)	−0.0594 (−0.0653, −0.0534)	0.0216 (0.018, 0.0253)
GLM-2SRI	0.0522 (0.0294, 0.0750)	−0.0753 (−0.116, −0.0343)	0.0085 (−0.0240, 0.0411)
LIV	−0.00973 (−0.0226, 0.00316)	0.0471 (−0.0829, 0.177)	0.0386 (0.00430, 0.0729)

2SRI, 2-stage residual inclusion; ES, emergency surgery; GLM, generalized linear model; LIV, local instrumental variables; NES, nonemergency surgery; QALYs, quality-adjusted life-years.

#### Subgroup analysis of cost-effectiveness of ES

**Figure 2:**
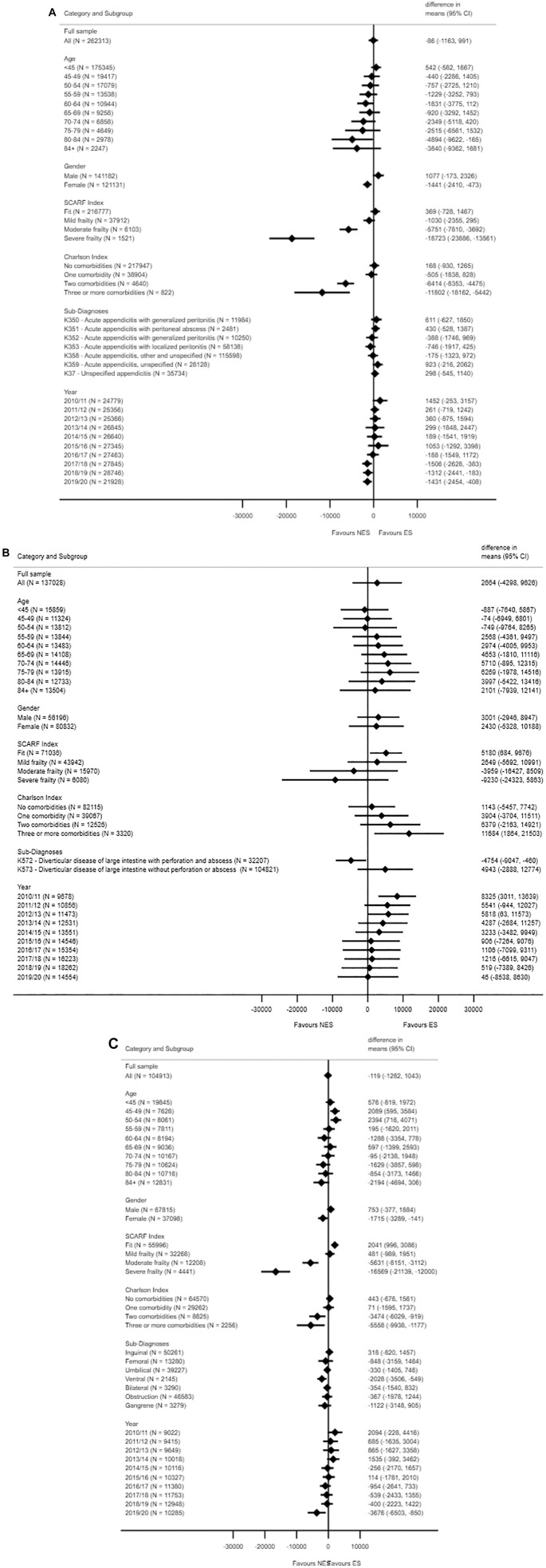
Estimated incremental net monetary benefit (INB) of emergency surgery (ES) versus nonemergency surgery (NES) strategies for acute appendicitis (A), diverticular disease (B), and abdominal wall hernia (C).(continued)

Figure 2 reports that beneath the overall LIV results, there is underlying heterogeneity in the INB estimates according to subgroup. For patients with acute appendicitis, ES appears less cost-effective for women, older patients, and those with 2 or 3 comorbidities. For each condition, ES is less cost-effective on average, according to increasing frailty levels. For example, for appendicitis, the estimated INBs for patients with moderate and severe frailty were −£5750 (−£7810, −£3692) and −£18,723 (−£23,886, −£13,561) versus £369 (−£728, £1467) for patients who were fit (see also Supplementary Figure S3).

#### Estimated individual-level effects of ES on costs and outcomes

**Figure 3:**
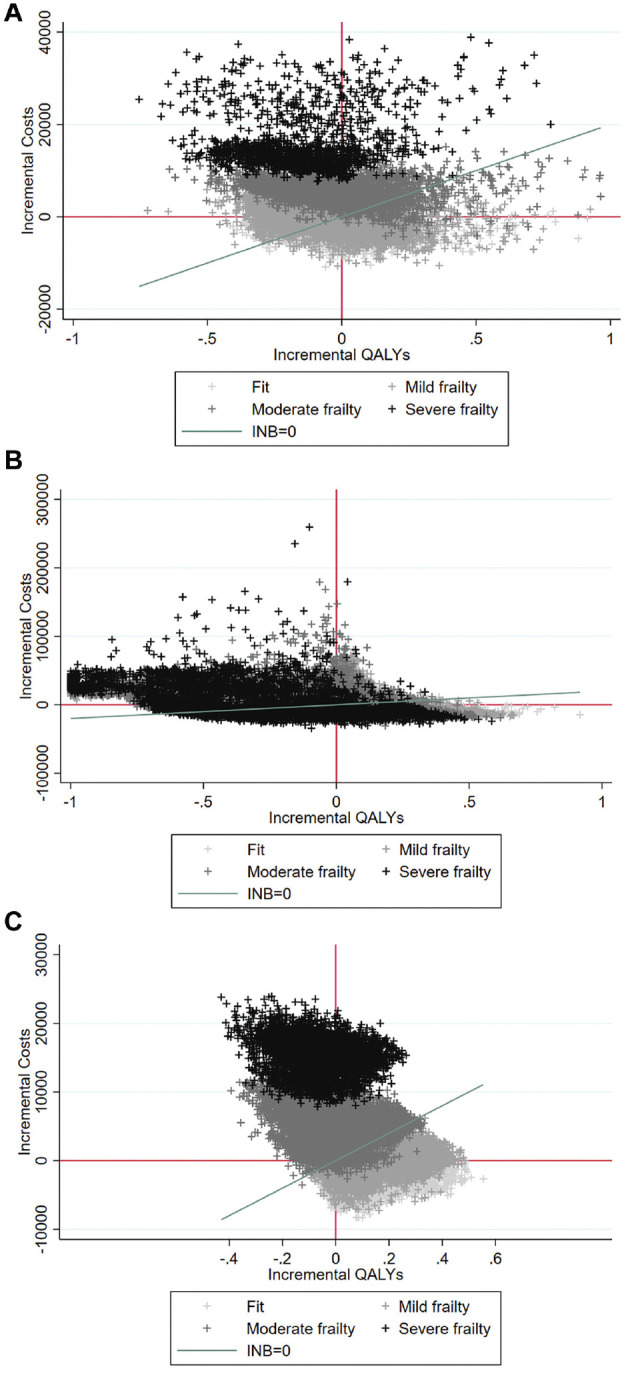
Cost-effectiveness plane of person-centered treatment effects on costs and quality-adjusted life-years (QALYs) for appendicitis (A), diverticular disease (B), and abdominal wall hernia (C). Person-centered treatment effects of emergency surgery on costs and for appendicitis, diverticular disease, and abdominal wall hernia, where each data point relates to 1 patient in the data set and each color to 1 band of the secondary care administrative records frailty (SCARF) index (fit is light gray, severe frailty is black).

Figure 3 reports the individual-level estimates of incremental costs and QALYs for the 3 conditions. Here, for illustration, the results are stratified by frailty level. For those with severe frailty, the proportion of patients for whom ES is estimated to be cost-effective is 0.0657% (appendicitis), 46.9% (diverticular disease), and 0.00% (hernia), whereas for patients who were fit, the corresponding proportions were 59.0% (appendicitis), 87.1% (diverticular disease), and 82.0% (hernia).

### Sensitivity Analyses

The overall results were robust to alternative assumptions (see Supplementary Table S9), including alternative definitions of hospital quality of care (SA1), higher (SA2) or lower (SA3) unit costs, and the use of linear interpolation for calculating QALYs (SA4). The extension to a 5-y time horizon resulted in a negative INB for appendicitis and diverticular disease (SA5), but the sample size was much reduced (∼50%), and the CIs surrounding the INB estimates over this extended time horizon were wide and, like the base case, included zero.

## Discussion

This article critically examines LIV methods for comparative effectiveness research using EHRs in the context of a CEA. We evaluate the cost-effectiveness of ES compared with NES alternatives for emergency admissions with common acute conditions. The IV design exploited the wide variations in ES rates across hospitals. The LIV method was chosen because it can address confounding and treatment effect heterogeneity, and provide cost-effectiveness estimates for the overall population as well as subpopulations of decision-making relevance, provided the models for the outcome and the treatment assignment are correctly specified. For diverticular disease, the results differed by method. Whereas the traditional approaches reported that, overall, ES was not cost-effective, the LIV approach reported that the overall results were highly uncertain. For appendicitis and hernia, all 3 approaches reported that the overall cost-effectiveness results were uncertain. For all 3 conditions, the LIV approach found heterogeneity in the cost-effectiveness estimates; in particular, ES was not cost-effective for patients with severe levels of frailty.

This article makes 3 important contributions to the literature. First, we add to the literature using IV methods for the evaluation of routinely provided interventions.^6,52,59–61^ In the EHR context, given that data are not collected for research purposes, finding a valid IV is especially challenging. This article exemplifies the use of EHRs to substantiate and assess the underlying assumptions of an IV design. For example, to address potential violations of the exclusion restriction, we examined whether the hospital’s TTO could minimize imbalances in measured covariates with balance plots and used “internal” (i.e., EHR data) and “external” (i.e., NELA^55–57^) information to adjust for the quality of acute care, and improve the plausibility of the exclusion restriction.

Second, this article constitutes a novel application of LIV to a CEA that uses EHR data. We show how EHRs can offer large sample sizes, enabling a CEA to provide precise cost-effectiveness results at the subgroup level, and to reflect the range of patients presenting in routine practice. This article also highlights major challenges of using EHR data for CEA, namely, unmeasured confounding and treatment effect heterogeneity. Although both IV methods considered rely on parametric assumptions and the validity of IV assumptions to address confounding, 2SRI can also fail to identify the ATE in the presence of essential heterogeneity.^62,63^ Hence, one interpretation of the differences between the estimates from 2SRI and LIV for patients with diverticular disease is that the estimated effects may differ between marginal patients and the overall population.^62^ For patients with diverticular disease, patients may well have been selected to receive ES according to measures that were not available in these EHR data, such as the severity of the disease, and so the 2SRI approach may have failed to validly identify the ATE.

Third, this article contributes to the limited previous literature evaluating the cost-effectiveness of ES for these common acute conditions. Some previous studies have also suggested that NES strategies can result in similar outcomes and costs for patients with appendicitis,^25,28,64^ whereas others have found NES to be more cost-effective than ES.^65^ Published RCTs evaluating ES strategies for acute diverticular disease have failed to recruit sufficiently large populations to explore heterogeneity across population subgroups^26^ and are nonexistent for acute hernia. Unlike previous studies,^25–28,65–72^ the ESORT study included large sample sizes (>100,000 for each condition) and subgroups (e.g., those with severe frailty) excluded from RCTs. These results can help decision makers identify subgroups for whom NES strategies are relatively cost-effective (e.g., patients with severe frailty), those for whom ES is more cost-effective (e.g., “fit” patients), and those for whom there is residual uncertainty and for whom further research may be most valuable.^73,74^

This study has several strengths. First, the study extended a previously validated IV approach by using large-scale EHR data.^50^ Second, the HES data, while having common features of EHR data (notably the potential for confounding and heterogeneity), were of generally high quality with baseline covariates, all-cause mortality, and resource use data available for ∼95% of patients. Third, the study considered 3 different conditions for which it was anticipated there would be heterogeneous treatment effects according to patient subgroups.

While we address some of the challenges of using EHRs for CEA, others remain. First, HRQoL data were not available from HES and had to be obtained from the literature. Granular baseline measures of disease severity (e.g., size of abscess) were not available to provide more nuanced subgroup definitions. Second, it is possible that coding errors within the HES data were incorporated into the estimates of cost and cost-effectiveness, although previous research found that costs estimated from HES data were very similar to those derived from medical records.^75^ Third, in common with any approach to address confounding, the implementation of the LIV methods made assumptions, in particular, that the relationships of the covariates and the IV, with both the treatment receipt and the outcomes, were correctly specified. Here, more flexible data-adaptive approaches may be helpful, although they have not yet been extended to this context. A further consideration is that subgroup analyses presented here represent the average estimated effect for individuals within the group rather than the causal effect of group membership per se. While the subgroups used here were prespecified within a statistical analysis plan, in other contexts spurious subgroup effects may be obtained by “*P*-hacking.”

This article identifies areas for future research. First, future research could build on this work by incorporating data-adaptive methods such as generalized random forests or lasso into the LIV estimation, or by using methods such as causal rule ensembles for exploring heterogeneity,^76^ while recognizing interactions among prognostic variables. Second, the methods used in this study could be extended to chronic diseases by considering other preference-based instruments (e.g., tendency to prescribe), or multiple IV such as genetic markers, which will raise new issues for the LIV approach. Finally, our results can be used to target future trials. For instance, for patients with abdominal wall hernia, there appears to be equipoise about the choice of strategy (∼50% in each comparison group). A future trial could collect granular information on patient subgroups, longitudinal HRQoL measures, and be nested within the EHR data to help ensure the results are directly applicable to clinical decision making.

## Supplemental Material

sj-docx-1-mdm-10.1177_0272989X221100799 – Supplemental material for Local Instrumental Variable Methods to Address Confounding and Heterogeneity when Using Electronic Health Records: An Application to Emergency Surgery Click here for additional data file. Supplemental material, sj-docx-1-mdm-10.1177_0272989X221100799 for Local Instrumental Variable Methods to Address Confounding and Heterogeneity when Using Electronic Health Records: An Application to Emergency Surgery by Silvia Moler-Zapata, Richard Grieve, David Lugo-Palacios, A. Hutchings, R. Silverwood, Luke Keele, Tommaso Kircheis, David Cromwell, Neil Smart, Robert Hinchliffe and Stephen O’Neill in Medical Decision Making
