# Supplementary material for: Local Instrumental Variable Methods to Address Confounding and Heterogeneity when Using Electronic Health Records: An Application to Emergency Surgery
Source: Med Decis Making. 2022 May 24;42(8):1010–26. doi: 10.1177/0272989X221100799 (PMC9583279; doi:10.1177/0272989X221100799)
Supplement: sj-docx-1-mdm-10.1177_0272989X221100799 – Supplemental material for Local Instrumental Variable Methods to Address Confounding and Heterogeneity when Using Electronic Health Records: An Application to Emergency Surgery [file sj-docx-1-mdm-10.1177_0272989X221100799.docx]

**Supplementary material for:**

**‘Local instrumental variable methods to address confounding and heterogeneity when using electronic health records: an application to emergency surgery.’**

**Contents**

**Supplemental notes**

S1 Costing methodology

S2 Calculation of QALYs

S3 Search for appropriate HRQoL scores and adjustment

S4 A Local instrumental variable (LIV) approach

S5 Accounting for hospital quality

**References**

**Supplemental tables**

S1 Definitions of populations and interventions

S2 Unit costs (£GBP 2019/20)

S3 Components of surgical sets

S4 Measures of resource use

S5 Literature search strategies for HRQoL scores

S6 HRQoL scores

S7 Selection of GLMs

S8 Common operative procedures

S9 Sensitivity analyses results

**Supplemental figures**

S1 Variation in TTO across hospitals

S2 Kaplan-Meier curves

S3 Forest plots for estimates of incremental costs, and QALYs

S4 HRQoL trajectory following initial emergency hospital admission and emergency hospital readmission(s) using immediate and linear interpolation

**Section 1: Costing methodology**

*Overview*

The main cost items for emergency surgery (ES) and non-emergency surgery (NES) strategies were the costs related to the length of stay in hospital, including the stay on general wards (bed-day costs) and on intensive care units (ICU). These costs covered both the index admission and any readmission (emergency or planned) up to one year, and used patient-level resource use data from HES, that linked information across all qualifying hospital admissions. The costs considered also included diagnostic and operative procedures. A micro-costing approach was adopted to calculate the costs of operative procedures in the index admission and readmissions.

*Costing operative procedures*

The study designated more common operative procedures as potential drivers of the incremental cost of ES versus NES, and therefore costed each of these procedures separately. For each of the comparison groups, the definition of ‘more common’ was a procedure with a prevalence that exceeded 1% in the index admission. This conservative definition of ‘more common’ was taken to reduce the risk of excluding important cost differences between the comparison groups. The 1% rule was deemed appropriate for also identifying ‘more common’ procedures in readmissions, which were costed using the same methods and assumptions used for procedures performed in the index admission. If multiple operative procedures met the criteria for ‘more common’, only one was costed. For each comparison group, we first considered procedures that would potentially qualify as ES (see Table S1 (D), and then considered operative procedures that did not meet the ES criteria (e.g. cholecystectomy) and then other non-operative procedures (e.g. catheterisation of bladder). For those admissions with procedures that did not meet the threshold for a common procedure because they were ‘low-volume’ (<1% for the comparison group in the initial admission) we did not include specific additional costs for the procedure per se, and only included the costs associated with bed-days and diagnostic tests^[[1]](#footnote-1)^.

Table S8 lists the most common operative procedures for each of the comparison groups for the three conditions.

To calculate the costs of the ‘common’ operative procedures, the expected durations of the operations, the number and grade of staff involved were informed by the literature and expert opinion (see Table S4). The use of disposables (e.g. reload staplers), equipment (e.g. imaging systems), surgical instruments (e.g. laparoscopic sets), and overheads were informed by expert opinion (see Table S4).

*Applying unit costs*

Each resource use item was valued using appropriate unit costs from recommended national sources (see Table S2 and S3). Direct personnel costs were calculated as the costs per hour of employing each grade of staff. The costs of overheads included costs of drugs, direct Central Sterile Supply Department (CSSD), as well as allocated costs (rent, property and equipment maintenance and cleaning costs, among others) associated with the provision of the procedure.^1^ Purchase prices of disposables, instruments and equipment for each procedure were retrieved from different sources, including the finance department of an NHS Trust hospital. The assignment of unit costs for each item took account of the expected number of times the item would be used over the lifetime, recognising any additional costs of a sterilisation process required to enable reuse.^2^ All unit costs were inflated to 2019/20 prices (£ GBP) using UK’s GDP deflator published by HM Treasury.^3^

To assess the sensitivity of the results to assumptions made about unit costs, the sensitivity analyses considered alternative scenarios. Specifically, the inclusion of the full unit costs of operative procedures risks double counting of those items (e.g. some consumables) that may be included within the overall costs per bed-day. Conversely, the exclusion of the ‘less common’ operative procedures for both comparison groups may have led to an underestimate of the absolute levels of costs for both groups. To investigate whether either standpoint would be likely to lead to a large inaccuracy in the estimation of incremental cost, the scenarios considered increases and decreases of by 10% (see Table S9).

**Section 2: Calculation of QALYs**

The cost-effectiveness analysis (CEA) was designed to report QALYs up to one year (base case) by combining individual-level survival data with appropriate health-related quality of life (HRQoL) estimates for acute appendicitis, diverticulitis and abdominal wall hernia, for ES and NES strategies.

For the base case analysis, HRQoL values were required at ‘baseline’, the time of the emergency admission, and at one-year follow-up. For survivors at one-year, it was assumed that patients’ HRQoL was reduced for the duration of the initial emergency hospital admission, and then following hospital discharge that the patient’s HRQoL level recovered immediately to the average HRQoL level reported in the literature at the one-year follow-up. For patients who had an emergency readmission recorded within the HES data, during the one-year follow-up, it was assumed that HRQoL at readmission reverted to the same level as that following the initial (index) emergency admission. It was also assumed that following hospital discharge the HRQoL levels reverted to those at one-year follow-up (see Figure S4 (A)). The assumption that HRQoL reverted to follow-up levels immediately after hospital discharge, was challenged in sensitivity analysis in which QALYs were instead calculated using linear interpolation between the index emergency admission and one-year follow-up (see Figure S4 (B)). For patients who died prior to one-year, a HRQoL score of zero was applied.

The approach to estimating QALYs therefore assumed that events that do not lead to emergency readmissions (e.g. planned surgery for recurrence), have minimal impact on the patient’s HRQoL, as suggested previously e.g. for hernia repairs in the elective setting, in^4,5^. It was also assumed that there is no differential effect on QALYs between the comparison groups, beyond the effect on one-year mortality, or the rate or duration of emergency readmissions, both of which were derived from the individual-level HES data. This was motivated by the limited availability of studies comparing HRQoL of ES to NES alternatives in the emergency setting. The QALY calculation recognised differences in HRQoL according to age and gender, by adjusting the general HRQoL values from the literature according to recommended age-gender weights derived from the general population (see Table S6). ^6,7^

**Section 3: Search for appropriate HRQoL scores and adjustment**

The approach to estimating QALYs required that appropriate HRQoL values were identified from a literature review. We undertook separate search strategies for each condition in MEDLINE (see Table S5). The criteria used to select the most appropriate source of HRQoL, recognised the specific requirements of the ESORT study,^8^ and were prioritised according to:

1. The study considered at least one intervention regarded as ES by the clinical panel.
2. The intervention was performed in the emergency (non-elective) setting.
3. The study evaluated HRQoL using the tool recommended by NICE in their methodological guidance, the EuroQoL 5-dimension (EQ-5D) instrument in its three-level (3L) version.^9^
4. The study evaluated HRQoL at baseline (i.e. pre-operatively), and at one-year from baseline.
5. The study was conducted in the UK, or in a country with similar demographics and healthcare system.
6. The study was conducted no earlier than ten years before the start date of the ESORT study (i.e. 2010).

Most of the studies that met these criteria compared different forms of ES, rather than ES versus NES strategies. We therefore applied the same HRQoL scores at baseline and one year to both comparison groups as outlined in Section 2, in keeping with the assumption noted above, that any differences in HRQoL between the comparison groups would be captured by differences in one-year mortality, and the rate and duration of emergency readmissions.

**Section 4: A Local instrumental variable (LIV) approach**

We consider the Neyman-Rubin potential outcomes framework^10,11^, where $Y_{1}=\mu_{1}(X_{O}, X_{U},\vartheta)$ and $Y_{0}=\mu_{0}(X_{O}, X_{U},\vartheta)$ are the potential outcomes under treatments 1 (ES) and 0 (NES) and $\Delta=Y_{1}-Y_{0}$ is the individual treatment effect, $X_{O}$ are observed characteristics (e.g. patient’s measured frailty), $X_{U}$ are unmeasured confounders (e.g. patient’s physiology) and $\vartheta$ captures any remaining unobserved random variation. The model for treatment assignment can be defined as $D^{*}=\mu_{D}(Z{,X}_{O})-U_{D}$ and $D=1 if D^{*}\geq0$, where $Z$ is a vector of instruments and $U_{D}$ captures $X_{U}$and any other unobserved variable that influences treatment selection. Here, the decision to assign the patient to ES (D=1) depends on their observed and unobserved characteristics and the tendency of their hospital to operate (i.e. the instrument). Following Heckman and Vytlacil^12,13^ and without loss of generality, this model can be re-written in terms of probabilities as $D^{*}=P(Z{,X}_{O})-V$, where $P(Z{,X}_{O})$ is the propensity for treatment, *V* reflects the degree to which unobserved variables discourage treatment and *V* is uniformly distributed between 0 and 1.

Note that under this model each complier (patient whose treatment status was altered by shifts in the level of IV) has some level of $Z$ at which they would have only just been assigned to ES. For any value of $Z$ below that “threshold”, the patient will remain in the comparator group. At this level of Z they would be in equipoise. These hypothetical patients in equipoise are referred to as marginal patients, since a marginal change in the IV is sufficient to alter their treatment assignment. Since the IV is assumed not to otherwise influence treatment, the change in outcomes attributable to this marginal change in the IV can be attributed to the change in treatment, thus we can identify the marginal treatment effect for these marginal patients. The Marginal Treatment Effect (MTE) can be defined as,

$$\Delta^{MTE}\left( x_{O}, v \right)=E(\Delta|X_{O}=x_{O},V=v)$$

The MTE is the most nuanced treatment effect parameter. Under regular IV assumptions, the Local instrumental variable (LIV) estimator can be used to estimate a series of MTEs. ^12–15^

$$\Delta^{MTE}\left( x_{O},p \right)=\frac{\partial E(Y_{1}-Y_{0}|X_{O}=x_{O}, P\left( z,x_{O} \right)=p)}{\partial p}$$

To estimate the MTEs, we (i) estimate the propensity score for ES for each individual using a probit model, including the measured confounders and the instrument (similar to the first stage in 2SRI, or 2SLS but using probit in place of linear regression), (ii) store the estimated propensity scores and make sure that there exists coverage for both treatment arms across all values from 0 to 1 (rounded to 0.01; or else drop values), (iii) estimate an outcome model (Generalised Linear Models (GLMs) here) on the covariates, propensity score and interactions of these using appropriate methods, (iv) take the derivative of the estimated outcome equation with respect to the estimated propensity score to obtain the MTE estimate. Then, MTEs can be aggregated into meaningful parameters of treatment effects such as the ATE or CATEs.

Basu showed that MTEs can also be used to derive person-centered treatment (PeT) effects ^16,17^. Note that the treatment assignment status provides some information on $V$ and $P(Z{,X}_{O})$ for each patient. For instance, a patient with low frailty who nonetheless is assigned to ES, is likely to have unmeasured characteristics encouraging ES (disease severity), that is the observed treatment assignment is informative about the range of unobserved confounders (and hence values of *V)* that are plausible for that patient.

For patients in the treatment group (D=1), the propensity to choose treatment based on X and Z must outweigh the propensity to choose the comparator strategy based on $U_{D}$, i.e. $P\left( z{,x}_{O} \right)>v$. For patients in the comparator strategy (D=0), the opposite is true. Hence,

$\Delta^{PeT}\left( x_{O},p,D \right)=E(Y_{1}-Y_{0}|X_{O}=x_{O},P\left( z,x_{O} \right)>v)$ for individuals with D=1

$\Delta^{PeT}\left( x_{O},p,D \right)=E(Y_{1}-Y_{0}|X_{O}=x_{O},P\left( z{,x}_{O} \right)<v)$ for individuals with D=0

The PeT effect averages MTEs with the same level of X and Z over those values of unobserved variables that are compatible with that patient’s treatment assignment. All the treatment effect parameters, including conditional average treatment effects (CATEs), can be derived by taking averages of PeT effects. This can be accomplished using the ‘petiv’ command in Stata.^17^ In short, we evaluate the MTE at different values of v, retaining only those that are consistent with the observed treatment decision given that patients observed characteristics and the level of the hospital’s tendency to operate (TTO, i.e. the IV) for their hospital, and then average these MTEs to obtain the PeT effect. The PeT effects can then be aggregated for the population of interest.

*Implementation for the CEA*

This LIV approach was implemented as follows: first, each patient’s propensity for ES was estimated according to their observed characteristics and the TTO using a probit model. Second, for each outcome (costs, QALYs), an appropriate GLM, determined by reference to the root mean squared error, was estimated relating the observed outcome to the individuals’ observed characteristics, and their propensity for ES, along with interactions between them. Next, the MTEs were obtained by considering the impact on outcomes of a marginal change in the propensity for ES. Third, numerical integration was used to obtain individual level treatment effect estimates recognising their actual treatment assignment as described in Basu^17^. After obtaining the effect estimates for Costs and QALYs, these were used to calculate the effect on Net Monetary Benefit (NMB), i.e. the incremental net monetary benefit (INB).

To obtain standard errors and confidence intervals, the steps above were bootstrapped 300 times (200 times for sensitivity analyses due to computational complexity), with all outcome models estimated within the same bootstrap to account for correlation between the cost and QALY endpoints. (For further details on the estimation steps, see^17^).

**Section 5: Accounting for hospital quality**

We derived proxy measures for the quality of acute care in managing emergency admissions. These proxy measure of quality of care, were defined by the rates of all-cause mortality and emergency readmissions up to 90 days for each hospital (base case). This information was reported for each condition for the 2009-2010 financial year, to provide baseline, time-invariant proxies for care quality in each hospital, and for the one year preceding each qualifying emergency hospital admission, to provide time-varying proxies for care quality. This allowed the study to adjust for time-constant differences in quality across hospitals, and those that differed over time. While an alternative approach would be to include hospital level fixed effects, these would only control for time invariant unobserved confounders, and would also remove much of the variation in TTO by hospital, thus weakening the IV substantially.

In sensitivity analyses, we consider ‘external’ measures of ‘quality of acute care’ by using hospital performance measures from the National Emergency Laparotomy Audit (NELA)^18–20^. Since data were not available from NELA for all years of the study, and definitions changed over time, we constructed an average (weighted by volume) using data from 2016, 2017 and 2018 for the following seven indicators of quality of peri-operative management for emergency laparotomy patients which we anticipate would capture the influence of any potential time invariant observed confounders associated with hospital quality:

1. Adjusted mortality rate
2. Proportion of patients in whom a risk assessment was documented preoperatively
3. Proportion of patients arriving in theatre within a time appropriate for the urgency of surgery
4. Proportion of patients with a calculated preoperative risk of death >5% for whom a consultant surgeon and anaesthetist were present in theatre
5. Admission to critical care when risk of death ≥5%
6. Unplanned returns to theatre
7. Unplanned returns to critical care

These variables were anticipated to control for a range of potential hospital-level unobserved confounders.

**References**

1. Information Services Division (ISD) Scotland. Theatres Costs-Detailed Tables - SFR 5.10., https://www.isdscotland.org/health-topics/hospital-care/operations-and-procedures/ (2019).

2. Ismail I, Wolff S, Gronfier A, et al. A cost evaluation methodology for surgical technologies. *Surg Endosc* 2015; 29: 2423–2432.

3. HM Treasury Department. Gross Domestic Product (GDP) deflators: user guide, https://assets.publishing.service.gov.uk/government/uploads/system/uploads/attachment_data/file/205904/GDP_Deflators_User_Guide.pdf (accessed 19 August 2021).

4. McCormack K, Wake B, Perez J, et al. Laparoscopic surgery for inguinal hernia repair: Systematic review of effectiveness and economic evaluation. *Health Technol Assess (Rockv)*; 9. Epub ahead of print 2005. DOI: 10.3310/hta9140.

5. Sharma P, Boyers D, Scott N, et al. The clinical effectiveness and cost-effectiveness of open mesh repairs in adults presenting with a clinically diagnosed primary unilateral inguinal hernia who are operated in an elective setting: systematic review and economic evaluation. *Health Technol Assess (Rockv)*; 19. Epub ahead of print 2015. DOI: 10.3310/hta19940.

6. Ara R, Brazier JE. Populating an economic model with health state utility values: Moving toward better practice. *Value Heal* 2010; 13: 509–518.

7. Ara R, Brazier J, Zouraq IA. The Use of Health State Utility Values in Decision Models. *Pharmacoeconomics* 2017; 35: 77–88.

8. ESORT Study Group. Emergency Surgery Or NoT (ESORT) study. *Study protocol*, https://www.lshtm.ac.uk/media/38711 (2020).

9. The National Institute for Health and Care. *Guide to the methods of technology appraisal 2013*, https://www.nice.org.uk/process/pmg9/resources/guide-to-the-methods-of-technology-appraisal-2013-pdf-2007975843781%0Ahttps://www.nice.org.uk/process/pmg9/chapter/foreword (2013).

10. Neyman J. On the application of probability theory to agricultural experiments. *Stat Sci* 1990; 5: 463–480.

11. Rubin D. B. Estimating causal effects of treatment in randomized and nonrandomized studies. *J Educ Psychol* 1974; 66: 688–701.

12. Heckman JJ, Vytlacil EJ. Local instrumental variables and latent variable models for identifying and bounding treatment effects. *Proc Natl Acad Sci U S A* 1999; 96: 4730–4734.

13. Heckman J, Vytlacil E. Structural equations, treatment effects and econometric policy evaluation. *Econometrica* 2005; 73: 669–738.

14. Bjorklund A, Moffitt R. The Estimation of Wage Gains and Welfare Gains in Self-Selection Models. *Rev Econ Stat* 1987; 69: 42.

15. Heckman J. Instrumental variables: A study of implicit behavioral assumptions used in making program evaluations. *J Hum Resour*; 32. Epub ahead of print 1997. DOI: 10.2307/146178.

16. Basu A. Estimating person-centered treatment (PeT) effects using instrumental variables: an application to evaluating prostate cancer treatments. *J Appl Econom* 2014; 29: 671–691.

17. Basu A. Person-centered treatment (PeT) effects: Individualized treatment effects using instrumental variables. *Stata J* 2015; 15: 397–410.

18. National Emergency Laparotomy Audit (NELA) Project Team. *Second patient report of the National emergency laparotomy audit*. London, 2016.

19. National Emergency Laparotomy Audit (NELA) Project Team. *Third patient report of the National emergency laparotomy audit*. London, www.nela.org.uk/reports (2017).

20. National Emergency Laparotomy Audit (NELA) Project Team. *Fourth patient report of the National emergency laparotomy audit*. London, 2018.

21. NHS Improvement. *NHS reference costs 2017/2018*. London, https://webarchive.nationalarchives.gov.uk/ukgwa/20200501111106/https://improvement.nhs.uk/resources/reference-costs/ (2018).

22. Curtis LA, Burns A. *Unit Costs of Health and Social Care 2019 | PSSRU*. Canterbury: Personal Social Services Research Unit, University of Kent, 2019.

23. Clement KD, Emslie K, Maniam P, et al. What is the Operative Cost of Managing Acute Appendicitis in the NHS: The Impact of Stump Technique and Perioperative Imaging. *World J Surg* 2020; 44: 749–754.

24. Javanmard-Emamghissi H, Boyd-Carson H, Hollyman M, et al. The management of adult appendicitis during the COVID-19 pandemic: an interim analysis of a UK cohort study. *Tech Coloproctol*. Epub ahead of print 2020. DOI: 10.1007/s10151-020-02297-4.

25. Heah S., Eu KW, Ho YH, et al. Abdominoperineal Resection for Palliation of Advanced Low Rectal Cancer. *Dis Colon Rectum* 1995; 5: 1313–17.

26. Teramoto A, Aoyama N, Ebisutani C, et al. Clinical importance of cold polypectomy during the insertion phase in the left side of the colon and rectum: a multicenter randomized controlled trial (PRESECT study). *Gastrointest Endosc* 2020; 91: 917–924.

27. Wu CC, Chueh SC, Tsai YC. Is contralateral exploration justified in endoscopic total extraperitoneal repair of clinical unilateral groin hernias - A Prospective cohort study. *Int J Surg* 2016; 36: 206–211.

28. Wilson M. Urinary catheterisation in the community: Exploring challenges and solutions. *Br J Community Nurs* 2016; 21: 492–496.

29. O’Leary DP, Walsh SM, Bolger J, et al. A Randomized Clinical Trial Evaluating the Efficacy and Quality of Life of Antibiotic-only Treatment of Acute Uncomplicated Appendicitis: Results of the COMMA Trial. *Ann Surg* 2021; 274: 240–247.

30. Thornell A, Angenete E, Bisgaard T, et al. Laparoscopic Lavage for Perforated Diverticulitis With Purulent Peritonitis. *Ann Intern Med* 2016; 164: 137–145.

31. Rutegård M, Gümüsçü R, Stylianidis G, et al. Chronic pain, discomfort, quality of life and impact on sex life after open inguinal hernia mesh repair: an expertise-based randomized clinical trial comparing lightweight and heavyweight mesh. *Hernia* 2018; 22: 411–418.

**Supplemental tables**

**Table S1: Definitions of populations (panel A and B) and interventions (C) for acute appendicitis, diverticular disease, and abdominal wall hernia**

**(A): List of International Classification of Diseases (ICD)-10 codes considered for inclusion criteria**

| Acute appendicitis  (N=268,144) | Diverticular disease (N=138,869) | Abdominal wall hernia (N=106,432) |
| --- | --- | --- |
| K35: Acute appendicitis | K57.0: Diverticular disease of small intestine with perforation and abscess | K40.0: Bilateral inguinal hernia, with obstruction, without gangrene |
| K35.2: Acute appendicitis with generalised peritonitis | K57.1: Diverticular disease of small intestine without perforation or abscess | K40.1: Bilateral inguinal hernia, with gangrene |
| K35.3: Acute appendicitis with localized peritonitis | K57.2: Diverticular disease of large intestine with perforation and abscess | K40.2: Bilateral inguinal hernia, without obstruction or gangrene |
| K35.8: Acute appendicitis, other and unspecified | K57.3: Diverticular disease of large intestine without perforation or abscess | K40.3: Unilateral or unspecified inguinal hernia, with obstruction, without gangrene |
| K37: Unspecified appendicitis | K57.4: Diverticular disease of both small and large intestine with perforation and abscess | K40.4: Unilateral or unspecified inguinal hernia, with gangrene |
|  | K57.5: Diverticular disease of both small and large intestine without perforation or abscess | K40.9: Unilateral or unspecified inguinal hernia, without obstruction or gangrene |
|  | K57.8: Diverticular disease of intestine, part unspecified, with perforation and abscess | K41.0: Bilateral femoral hernia, with obstruction, without gangrene |
|  | K57.9: Diverticular disease of intestine, part unspecified, without perforation or abscess | K41.1: Bilateral femoral hernia, with gangrene |
|  |  | K41.2: Bilateral femoral hernia, without obstruction or gangrene |
|  |  | K41.3: Unilateral or unspecified femoral hernia, with obstruction, without gangrene |
|  |  | K41.4: Unilateral or unspecified femoral hernia, with gangrene |
|  |  | K41.9: Unilateral or unspecified femoral hernia, without obstruction or gangrene |
|  |  | K42.0: Umbilical hernia with obstruction, without gangrene |
|  |  | K42.1: Umbilical hernia with gangrene |
|  |  | K42.9: Umbilical hernia without obstruction or gangrene |
|  |  | K43.0: Incisional hernia with obstruction, without gangrene |
|  |  | K43.1: Incisional hernia with gangrene |
|  |  | K43.2: Incisional hernia without obstruction or gangrene |
|  |  | K43.3: Parastomal hernia with obstruction, without gangrene |
|  |  | K43.4: Parastomal hernia with gangrene |
|  |  | K43.5: Parastomal hernia without obstruction or gangrene |
|  |  | K43.6: Other and unspecified ventral hernia with obstruction, without gangrene |
|  |  | K43.7: Other and unspecified ventral hernia with gangrene |
|  |  | K43.9: Other and unspecified ventral hernia without obstruction or gangrene |

**(B): List of exclusion criteria**

| Acute appendicitis  (N=268,144) | Diverticular disease (N=138,869) | Abdominal wall hernia (N=106,432) |
| --- | --- | --- |
| Pregnancy | None | Pregnancy |
| Appendiceal cancer |  | Ischaemia |
|  |  | Cancer |

**(C): Definition of ‘emergency surgery’ and time window**

|  | Acute appendicitis (N=268,144) | Diverticular disease (N=138,869) | Abdominal wall hernia (N=106,432) |
| --- | --- | --- | --- |
| Procedures defined as ‘emergency surgery’ | See Table S1 (D) | See Table S1 (D) | See Table S1 (D) |
| Common procedures excluded from definition of ‘emergency surgery’ | *Unspecified other excision of appendix | Image controlled percutaneous drainage | None |
| Threshold for a procedure in the index admission to be ‘emergency surgery’ | 7 days | Any time | 3 days |
| Threshold for a procedure in a readmission to be ‘emergency surgery’ | 7 days | 14 days | 3 days |

*Further OPCS Classification of Interventions and Procedures (OPCS-4) codes were added to the list of ES procedures after the clinical panel exercise. For appendicitis (following review of coding use by hospital): H029 Unspecified other excision of appendix. For abdominal wall hernia (following inclusion of umbilical hernia as a diagnosis and for consistency with other hernia types): T241 Repair of umbilical hernia using insert of natural material, T248 Other specified primary repair of umbilical hernia, T971 Repair of recurrent umbilical hernia using insert of natural material, T973 Repair of recurrent umbilical hernia using sutures, T978 Other specified repair of recurrent umbilical hernia, T979 Unspecified repair of recurrent umbilical hernia. See Table S1 (D) for full list of OPCS codes defined as emergency surgery.

**(D): Full list of OPCS codes defined as emergency surgery**

| Acute appendicitis  (N=268,144) | Diverticular disease (N=138,869) | Abdominal wall hernia (N=106,432) |
| --- | --- | --- |
| H011: Emergency excision of abnormal appendix and drainage  H012: Emergency excision of abnormal appendix NEC  H018: Other specified emergency excision of appendix  H019: Unspecified emergency excision of appendix  H029: Unspecified other excision of appendix  H031: Drainage of abscess of appendix  H032: Drainage of appendix  H071: Right hemicolectomy and end to end anastomosis of ileum to colon  H072: Right hemicolectomy and side to side anastomosis of ileum to transverse colon  H073: Right hemicolectomy and anastomosis NEC  H074: Right hemicolectomy and ileostomy HFQ  T342: Open drainage of pelvic abscess  T343: Open drainage of abdominal abscess  H013: Emergency excision of normal appendix  T463: Irrigation of peritoneal cavity  H062: Extended right hemicolectomy and anastomosis of ileum to colon  H078: Other specified other excision of right hemicolon  T468: Other specified other drainage of peritoneal cavity | H091: Left hemicolectomy and end to end anastomosis of colon to rectum  H092: Left hemicolectomy and end to end anastomosis of colon to colon  H093: Left hemicolectomy and anastomosis  H094: Left hemicolectomy and ileostomy  H095: Left hemicolectomy and exteriorisation of bowel NEC  H101: Sigmoid colectomy and end to end anastomosis of ileum to rectum (0.03%)  H102: Sigmoid colectomy and anastomosis of colon to rectum  H103: Sigmoid colectomy and anastomosis  H104: Sigmoid colectomy and ileostomy  H105: Sigmoid colectomy and exteriorisation of bowel  H113: Colectomy and anastomosis NEC (0.01%)  H114: Colectomy and ileostomy  H115: Colectomy and exteriorisation of bowel  H152: End colostomy  H158: Other specified other exteriorisation of colon  H333: Anterior resection of rectum and anastomosis of colon to rectum using staples  H334: Anterior resection of rectum and anastomosis NEC  H335: Rectosigmoidectomy and closure of rectal stump and exteriorisation of bowel  H336: Anterior resection of rectum and exteriorisation of  T342: Open drainage of pelvic abscess (0.09%) 33 | T201: Primary repair of inguinal hernia using insert of natural material  T202: Primary repair of inguinal hernia using insert of prosthetic material  T203: Primary repair of inguinal hernia using sutures  T204: Primary repair of inguinal hernia and reduction of sliding hernia  T208: Other specified primary repair of inguinal hernia  T209: Unspecified primary repair of inguinal hernia  T211: Repair of recurrent inguinal hernia using insert of natural material  T212: Repair of recurrent inguinal hernia using insert of prosthetic material  T213: Repair of recurrent inguinal hernia using sutures  T218: Other specified repair of recurrent inguinal hernia  T219: Unspecified repair of recurrent inguinal hernia  T221: Primary repair of femoral hernia using insert of natural material  T222: Primary repair of femoral hernia using insert of prosthetic material  T223: Primary repair of femoral hernia using sutures  T228: Other specified primary repair of femoral hernia  T229: Unspecified primary repair of femoral hernia  T231: Repair of recurrent femoral hernia using insert of natural material  T232: Repair of recurrent femoral hernia using insert of prosthetic material  T233: Repair of recurrent femoral hernia using sutures  T239: Unspecified repair of recurrent femoral hernia  T241: Repair of umbilical hernia using insert of natural material  T242: Repair of umbilical hernia using insert of prosthetic material  T243: Repair of umbilical hernia using sutures  T248: Other specified primary repair of umbilical hernia  T249: Unspecified primary repair of umbilical hernia  T271: Repair of ventral hernia using insert of natural material  T272: Repair of ventral hernia using insert of prosthetic material  T273: Repair of ventral hernia using sutures  T278: Other specified repair of other hernia of abdominal wall  T279: Unspecified repair of other hernia of abdominal wall  T288: Other specified other repair of anterior abdominal wall  G762: Open relief of strangulation of ileum  G763: Open relief of obstruction of ileum NEC  H176: Open relief of obstruction of colon NEC  T251: Primary repair of incisional hernia using insert of natural material  T971: Repair of recurrent umbilical hernia using insert of natural material  T972: Repair of recurrent umbilical hernia using insert of prosthetic material  T973: Repair of recurrent umbilical hernia using sutures  T978: Other specified repair of recurrent umbilical hernia  T979: Unspecified repair of recurrent umbilical hernia  T981: Repair of recurrent ventral hernia using insert of natural material  T982: Repair of recurrent ventral hernia using insert of prosthetic material  T983: Repair of recurrent ventral hernia using sutures  T989: Unspecified repair of recurrent other hernia of abdominal wall  T252: Primary repair of incisional hernia using insert of prosthetic material  T253: Primary repair of incisional hernia using sutures  T258: Other specified primary repair of incisional hernia  T259: Unspecified primary repair of incisional hernia 44  T261: Repair of recurrent incisional hernia using insert of natural material  T262: Repair of recurrent incisional hernia using insert of prosthetic material  T263: Repair of recurrent incisional hernia using sutures  T268: Other specified repair of recurrent incisional hernia  T269: Unspecified repair of recurrent incisional hernia  T318: Other specified other operations on anterior abdominal wall |

**Table S2: Unit costs (£GBP 2019/20) for potential cost drivers**

| Item | Unit | Unit cost (£GBP) | Source, definitions and assumptions |
| --- | --- | --- | --- |
| Inpatient stay |  |  |  |
| *General ward* | Day | 347 | NHS Reference costs 2017/18. Weighted average of FD05A and FD05B (NEL_XS)^21^ |
| *ICU ward* |  |  |  |
| Level 2 ICU | Day | 1,188 | NHS Reference costs 2017/18. XC06Z: 1 organ supported (adult critical care)^21^ |
| Level 3 ICU | Day | 1,886 | NHS Reference costs 2017/18. Weighted average of XC01Z-XC05Z. 2 to 6+ organs supported (adult critical care)^21^ |
| Diagnostic procedures |  |  |  |
| *More common diagnostic procedures for acute appendicitis* | | | |
| Computed tomography | Procedure | 83 | NHS Reference costs 2017/18. RD20A: Computerised Tomography Scan of One Area, without Contrast, 19 years and over (IMAG)^21^ |
| Unspecified diagnostic endoscopic examination of colon | Procedure | 206 | NHS Reference costs 2017/18. FE31Z: Diagnostic Colonoscopy with Biopsy, 19 years and over (NES). Mean bed-day costs of general ward subtracted to avoid double-counting^21^ |
| Fibreoptic endoscopic examination of upper gastrointestinal tract and biopsy of lesion of upper gastrointestinal tract | Procedure | 197 | NHS Reference costs 2017/18. FE21Z: Diagnostic Endoscopic Upper Gastrointestinal Tract Procedures with Biopsy, 19 years and over (NES). Mean bed-day costs of general ward subtracted to avoid double-counting^21^ |
| Diagnostic fibreoptic endoscopic examination of colon and biopsy of lesion of colon | Procedure | 277 | NHS Reference costs 2017/18. FE31Z: Diagnostic Colonoscopy with Biopsy, 19 years and over (NES). Mean bed-day costs of general ward subtracted to avoid double-counting^21^ |
| Computed tomography of head | Procedure | 83 | NHS Reference costs 2017/18. RD20: Computerised Tomography Scan of One Area, without Contrast, 19 years and over (IMAG)^21^ |
| *More common diagnostic procedures for diverticular disease* | | | |
| Computed tomography | Procedure | 83 | NHS Reference costs 2017/18. RD20: Computerised Tomography Scan of One Area, without Contrast, 19 years and over (IMAG)^21^ |
| Unspecified diagnostic endoscopic examination of lower bowel using fibreoptic sigmoidoscope | Procedure | 143 | NHS Reference costs 2017/18. FE35Z: Diagnostic Flexible Sigmoidoscopy, 19 years and over (NES). Mean bed-day costs of general ward subtracted to avoid double-counting^21^ |
| Unspecified diagnostic endoscopic examination of colon | Procedure | 206 | NHS Reference costs 2017/18. FE32Z: Diagnostic Colonoscopy, 19 years and over (NES). Mean bed-day costs of general ward subtracted to avoid double-counting^21^ |
| Unspecified diagnostic fibreoptic endoscopic examination of upper gastrointestinal tract | Procedure | 277 | NHS Reference costs 2017/18. FE31Z: Diagnostic Colonoscopy with Biopsy, 19 years and over (NES). Mean bed-day costs of general ward subtracted to avoid double-counting^21^ |
| Diagnostic endoscopic examination of lower bowel and biopsy of lesion of lower bowel using fibreoptic sigmoidoscope | Procedure | 205 | NHS Reference costs 2017/18. FE34Z: Diagnostic Flexible Sigmoidoscopy with Biopsy, 19 years and over (NES). Mean bed-day costs of general ward (see above) subtracted to avoid double-counting^21^ |
| *More common diagnostic procedures for hernia* | | | |
| Computed tomography | Procedure | 83 | NHS Reference costs 2017/18. RD20A: Computerised Tomography Scan of One Area, without Contrast, 19 years and over (IMAG)^21^ |
| Transthoracic echocardiography | Procedure | 101 | NHS Reference costs 2017/18. RD51C: Simple Echocardiogram, 5 years and under (IMAG)^21^ |
| Computed tomography of abdomen | Procedure | 83 | NHS Reference costs 2017/18. RD20: Computerised Tomography Scan of One Area, without Contrast, 19 years and over (IMAG)^21^ |
| Computed tomography of head | Procedure | 83 | NHS Reference costs 2017/18. RD20A: Computerised Tomography Scan of One Area, without Contrast, 19 years and over (IMAG)^21^ |
| Diagnostic endoscopic examination of peritoneum | Procedure | 404 | NHS Reference costs 2017/18. FE31Z: Diagnostic Colonoscopy with Biopsy, 19 years and over (NES). Mean bed-day costs of general ward subtracted to avoid double-counting^21^ |
| Operative procedures |  |  |  |
| *Staff input* | | | |
| Consultant surgeon | Minute | 1.8 | 2019 Unit costs of Health and Social Care (PSSRU). Section 14. Cost per working hour: consultant: surgical^22^ |
| Anaesthesiologist | Minute | 1.8 | 2019 Unit costs of Health and Social Care (PSSRU). Section 14. Cost per working hour: consultant: medical^22^ |
| Consultant radiologist | Minute | 1.8 | 2019 Unit costs of Health and Social Care (PSSRU). Section 14. Cost per working hour: consultant: medical^22^ |
| Registrar – surgery | Minute | 0.8 | 2019 Unit costs of Health and Social Care (PSSRU). Section 14. Cost per working hour: registrar^22^ |
| Registrar – anaesthesiology | Minute | 0.8 | 2019 Unit costs of Health and Social Care (PSSRU). Section 14. Cost per working hour: registrar^22^ |
| Registrar – radiology | Minute | 0.8 | 2019 Unit costs of Health and Social Care (PSSRU). Section 14. Cost per working hour: registrar^22^ |
| Nurse – Band 5 | Minute | 0.6 | 2019 Unit costs of Health and Social Care (PSSRU). Section 13. Cost per working hour: band 5 – hospital-based nurse^22^ |
| Nurse – Band 6 | Minute | 0.8 | 2019 Unit costs of Health and Social Care (PSSRU). Section 13. Cost per working. hour: band 6 – hospital-based nurse^22^ |
| Operating department practitioner | Minute | 0.8 | 2019 Unit costs of Health and Social Care (PSSRU). Section 13. Assumed same cost as cost per working hour of band-6 hospital-based nurse (Chapter 13)^22^ |
| *Overhead costs* | | | |
| Operating room | Minute | 5.4 | Includes direct drug and CSSD costs as well allocated costs (other staff; property and equipment maintenance; domestics and cleaning; heat, light and power; rent and rates; purchases of furniture, fittings and equipment (non-capital charge) and others). Weighted average of 43 hospitals in Scotland^1^. |
| *Reusable instruments and equipment* | | | |
| Laparoscopic colorectal set | Procedure | 39.2 | Manufacturer. See Table S3 for full list of components. Total purchase cost is £3,112. Number of uses is 2,750. Final cost includes sterilisation cost following at £0.8 cost per instrument used^2^ |
| Main laparoscopic set | Procedure | 36.8 | Manufacturer. See Table S3 for full list of components. Total purchase cost is £2,511. Assumed number of uses is 2,750. Final cost includes sterilisation cost following at £0.8 cost per instrument used^2^ |
| Major general set | Procedure | 39.2 | Manufacturer. See Table S3 for full list of components. Total purchase cost is £2,744. Assumed number of uses is 2,750. Final cost includes sterilisation cost following at £0.8 cost per instrument used^2^ |
| Minor general set | Procedure | 32.8 | Manufacturer. See Table S3 for full list of components. Total purchase cost is £1,417. Assumed number of uses is 2,750. Final cost includes sterilisation cost following at £0.8 cost per instrument used^2^ |
| Endoscopic polypectomy set | Procedure | 16.2 | Manufacturer. Includes endoscopic forceps, snare and endoscopic clips. Final cost includes sterilisation cost following at £0.8 cost per instrument used. ^2^ Unit cost calculated assuming number of uses is 4400 (except for snare and clips which are assumed to be disposable). |
| Telescope and stack | Procedure | 15.2 | Manufacturer. Includes stack, scope (Precision ideal eyes 10mm 30°, HD autoclavable Laparoscope 33cm), tray and cable (fibreoptic cable 5.0mm x 10 ft. (3.05m)). Purchase cost of stack and stack are £68,760 and £2,334, respectively. Unit cost calculated assuming number of uses is 4400. |
| Ultrasound system | Procedure | 1.5 | Manufacturer. Purchase cost of ultrasound system is £7,132. Unit cost calculated assuming number of uses is 4400. |
| *Disposables* | | | |
| Laparoscopic linear stapler | Procedure | 262 | Manufacturer. Linear Cutter 75mm. 1 is assumed to be used per procedure. |
| Stapler reload | Procedure | 36.5 | Manufacturer. Reload linear cutter, blue, 75mm. Purchase cost of £465.61 per box of 12. 1 is assumed to be used per procedure. |
| Endoloop ligature | Procedure | 56.9 | Manufacturer. Endoloop Ethicon. 3 are assumed to be used per procedure^23^ |
| Biosynthetic mesh | Procedure | 61.1 | Manufacturer. [Sutumed Polipropilene Non-absorbable Hernia Mesh 12" X 12"](https://www.google.com/aclk?sa=L&ai=DChcSEwiy8fiEnNHyAhWNgVAGHRraBq4YABAvGgJkZw&sig=AOD64_29bODdCw3aMG70X1pVDHfKDIMKGA&ctype=5&q=&ved=0ahUKEwiFifSEnNHyAhXTSsAKHaSUCdoQ2CkI6QU&adurl=). 1 is assumed to be used per procedure. |
| Abdominal drain set | Procedure | 18.2 | Manufacturer. Set includes 1000mL drainage bag, catheter valve cap, slide clamp, tape strips and wipe. Purchase cost £36.5 per box of 2. |
| Foyle catheterisation kit | Procedure | 9.4 | Manufacturer. Catheterisation Set 16fr Foley and extras. Includes a 16fr Foley catheter, a 500ml leg-bag, 2000ml bedside drainage bag, sterile syringe and lube. |

CSSD: Central sterile services department, ICU: intensive care unit, NHS: National Health Service.

**Table S3: Full list of components of surgical sets considered in cost analysis**

| Laparoscopic colorectal set | Main laparoscopic set | Major general set | Minor general set |
| --- | --- | --- | --- |
| Aesculap dorsey forcep 4 parts  Anti-tamper tags  B p handle no 3  B p handle no 4  Babcock tissue forcep long  Babcock tissue forcep short  Bottom tray  Container  Container identification label  Diathermy dissecting forcep mcindoe  Diathermy quiver  Diathermy quiver long + black end cap  Dissecting forcep debakey 6"  Dissecting forcep debakey 8"  Dissecting forcep gillies toothed  Doyen intestinal clamp curved  Dunhill artery forcep  Dyball retractor  Filter and retaining clip  Grasping forcep + ratchet with connector (pm 109)  Hasson 12mm (3 parts) ea12nh send disassembled  Heiss artery forcep  Insulated hook with connector  Ireusable cannula 12mm  Lanes dissecting forcep (1-2 teeth)  Laparoscopic diathermy lead (8mm bovie)  Littlewoods tissue forcep  Maryland f/cep no ratchet with connector (pm 102)  Massons needle holder  Monopolar diathermy lead pin fitting  Needle holder mayo hegar  Nelson robert scissors  Parker kerr intestinal clamp straight  Retractor langenbeck medium  Retractor langenbeck small  Retractor morris medium  Roberts artery forcep  Scissor mayo straight  Scissor mcindoe curved  Sh/sh scissor  Sponge holder rampley  Threaded cannula 5mm (2 parts)  Top tray  Trayliner  Trocar blunt tip 12mm  Trocar pencil point 12mm  Trocar pencil point 5mm  Trocar sharp tip 5mm  Waughs diathermy dissecting forcep | Anti-tamper tags  B p handle no 3  B p handle no 4  Bottom tray  Container  Container identification label  De-jardin stone forcep  Diathermy dissecting forcep mcindoe  Diathermy quiver long + black end cap  Dissecting forcep debakey 6"  Dissecting forcep gillies toothed  Dunhill artery forcep  Eragon ratchet handle - do not assemble to forcep  Filter and retaining clip  Grasping forcep + ratchet with connector (pm 109)  Hassan 10mm (2 parts+10mm clear seal)  Insulated hook with connector  Lanes dissecting forcep (1-2 teeth)  Laparoscopic diathermy lead (8mm bovie)  Littlewoods tissue forcep  Maryland forcep no ratchet with connector (pm 102)  Mesh basket with lid  Modular monopolar forcep (johan) sn 8393.184 2 parts  Monopolar diathermy lead pin fitting  Myoma forcep + ratchet with connector (pm 117)  Needle holder crilewood  Needle holder mayo hegar  Pike mouth forcep + ratchet with connector (pm 107)  Retractor langenbeck medium  Retractor langenbeck small  Reusable cannula 10mm  Reusable cannula 12mm  Scissor mayo straight  Scissor mcindoe curved  Sh/sh scissor  Spencer wells artery forceps 7" curved  Sponge holder rampley  Threaded cannula 5mm (2 parts)  Top tray  Towel clip small  Trayliner  Trocar blunt tip 10mm  Trocar pencil point 12mm  Trocar pencil point 5mm  Trocar sharp tip 5mm  Wash basket | B p handle no 4  B p handle no 5  Babcock tissue forcep 6 1/2"  Babcock tissue forcep 8"  Balfour self-retaining retractor (see remarks)  Deaver retractor, broad  Deaver retractor, narrow  Diathermy dissecting forcep mcindoe  Diathermy quiver  Disposable green tray wrap 120 x 150  Dissecting forcep debakey 6"  Dissecting forcep debakey 8"  Dissecting forcep debakey 9 1/2"  Dissecting forcep gillies toothed  Dissecting forcep non toothed 5"  Doyen intestinal clamp curved  Doyen intestinal clamp straight  Dunhill artery forcep  Dyball retractor  Heiss artery forcep  Lahey artery forceps  Lanes dissecting forcep (1-2 teeth)  Lang stevenson intestinal clamps  Littlewoods tissue forcep  Massons needle holder  Mayo pin holding next 1 item  Mayo pin holding next 3 items  Mayo pin holding next 4 items  Mayo pin holding next 6 items  Monopolar diathermy lead pin fitting  Moynihan cholecystectomy clamp  Needle holder mayo hegar 7 1/4"  Needle holder mayo hegar 8 1/2"  Nelson robert scissors  Parker kerr intestinal clamp curved  Parker kerr intestinal clamp straight  Retractor langenbeck medium  Retractor morris large  Roberts artery forcep  Scissor mayo curved  Scissor mayo straight 5 3/4"  Scissor mcindoe curved  Sh/sh scissor  Soaker sheet to be placed under basket/tray  Sponge holder rampley  Styles tissue forcep  Trayliner  Wash basket  Waughs diathermy dissecting forcep | Artery forcep mosquito curved  B p handle no 3  B p handle no 4  Babcock tissue forcep  Catspaw retractor  Diathermy dissecting forcep mcindoe  Diathermy quiver  Disposable blue tray wrap 120 x 150  Disposable green tray wrap 120 x 150  Dissecting forcep debakey 6"  Dissecting forcep gillies toothed  Dunhill artery forcep  Heiss artery forcep  Lahey artery forceps  Lanes dissecting forcep (1-2 teeth)  Littlewoods tissue forcep  Mayo pin holding next 2 items  Mayo pin holding next 3 items  Mayo pin holding next 4 items  Meyarding finger retractor  Monopolar diathermy lead pin fitting  Needle holder crilewood  Needle holder mayo hegar  Poirers/allis tissue forcep  Retractor langenbeck medium  Retractor langenbeck small  Retractor morris medium  Retractor self-retaining travers  Retractor self-retaining west  Scissor kilner curved  Scissor mayo curved  Scissor mayo straight 5 3/4"  Scissor mcindoe curved  Sh/sh scissor  Soaker sheet to be placed under basket/tray  Spencer wells artery forceps 7" curved  Spencer wells artery forceps 8" straight  Sponge holder rampley  T.o.e. dissecting forcep  Trayliner  Wash basket |

**Table S4: Resource use categories for operative procedures in emergency surgery (ES) window**

|  |  | Acute appendicitis | | Diverticular disease | | Abdominal wall hernia | |
| --- | --- | --- | --- | --- | --- | --- | --- |
|  |  | **ES (N=247,506)** | **NES (N=20,638)** | **ES (N=15,772)** | **NES (N=123,097)** | **ES (N=62,559)** | **NES (N=43,873)** |
| Most common operative procedures in each arm in ES window | - | Emergency excision of abnormal appendix | Interval appendicectomy | Rectosigmoidectomy and closure of rectal stump and exteriorisation of bowel | Fibreoptic endoscopic snare resection of lesion of colon | Primary repair of inguinal hernia using insert of prosthetic material | Unspecified urethral catheterisation of bladder |
| Time in theatre in minutes (source) | Literature/  expert opinion | 70 (^24^) | 70 (^24^) | 135 (^25^)* | 25 (^26^) | 60 (^27^) | 15 (^28^) |
| Staffing levels | Expert opinion | S1 | S1 | S1 | S1 | S1 | S2 |
| Instruments | Expert opinion | Main laparoscopic set | Main laparoscopic set | Major general set | Endoscopic polypectomy set | Minor general set | - |
| Equipment | Expert opinion | Laparoscope, cable and tray | Laparoscope, cable and tray | - | Laparoscope, cable and tray | - | - |
| Main disposables | Expert opinion | Three loops for closure of the appendiceal stump | Three loops for closure of the appendiceal stump | Laparoscopic linear stapler and reload | - | Biosynthetic mesh | Foyle catheterisation kit |

Table includes exemplar data for most common operative procedures in ES window. Resource use for all other operative procedures was calculated considering the same categories. *If the procedure appeared with operative codes for loop colostomy, other specified other exteriorisation of colon, or unspecified other exteriorisation of colon, the duration was assumed to be 205 minutes. S1 considered: 1 consultant surgeon, 1 registrar surgeon, 2 band 5 nurses, 1 band 6 nurse, 1 operating department practitioner, 1 consultant anaesthetist, 1 registrar anaesthetist. S2 considered 1 band 5 nurse.

**Table S5: Search strategies for HRQoL data**

**(A): Appendicitis**

Database: Ovid MEDLINE(R) ALL <1946 to August 19, 2021>

Search Strategy:

--------------------------------------------------------------------------------

1 *appendicitis/ (16067)

2 *appendectomy/ (6399)

3 appendic*.ti,ab. (33073)

4 appendec*.ti,ab. (10129)

5 emergency+surgery*.ti,ab. (9412)

6 emergency+appendectomy*.ti,ab. (153)

7 non-operative+manag*.mp. (1888)

8 conservative+manag*.mp. (16648)

9 antibiotic*.ti,ab. (360099)

10 antibiotic+adj+therapy.ti,ab. (0)

11 Anti-Bacterial+Agents/tu (135940)

12 Watchful+wait$.tu. (0)

13 delayed+surg$.ti,ab. (2186)

14 trial.ti,ab. (657219)

15 RCT.ti,ab. (24987)

16 randomi#ed+controlled+trial.pt. (541163)

17 controlled+clinical+trial.pt. (94345)

18 case+control+stud$.ti,ab. (113048)

19 cross-sectional+stud$.ti,ab. (197037)

20 cohort+stud$.ti,ab. (244943)

21 observational+stud$.ti,ab. (126477)

24 Economic+evaluation.ti,ab. (9983)

25 EuroQol-5+Dimension.ti,ab. (670)

26 "EQ-5D".ab. (9451)

27 or/1-2 (19106)

28 or/3-13 (496587)

29 and/27-28 (16860)

30 or/14-24 (1682168)

31 and/29-30 (1350)

32 or/25-26 (9720)

33 and/31-32 (4)

--------------------------------------------------------------------------------

**(B): Diverticular disease**

Database: Ovid MEDLINE(R) ALL <1946 to September 24, 2021>

Search Strategy:

--------------------------------------------------------------------------------

1 *diverticulitis/ (2667)

2 *Diverticulum/ (8202)

3 Diverticul*.mp. (33728)

4 emergency+surgery*.ti,ab. (9470)

5 Drainage*.ti,ab. (97292)

6 Lavage*.ti,ab. (52937)

7 Percutaneous+drainage*.ti,ab. (4232)

8 sigmoidectomy*.ti,ab. (1089)

9 colectomy*.mp. (24998)

10 conservative+manag*.mp. (16769)

11 antibiotic*.ti,ab. (362483)

12 antibiotic+adj+therapy.ti,ab. (0)

13 Anti-Bacterial+Agents/tu (136787)

14 Watchful+wait$.tu. (0)

15 delayed+surg$.ti,ab. (2207)

16 trial.ti,ab. (662690)

17 RCT.ti,ab. (25317)

18 randomi#ed+controlled+trial.pt. (544498)

19 controlled+clinical+trial.pt. (94426)

20 case+control+stud$.ti,ab. (113845)

21 cross-sectional+stud$.ti,ab. (200110)

22 cohort+stud$.ti,ab. (248537)

23 observational+stud$.ti,ab. (128258)

24 Economic+evaluation.ti,ab. (10055)

25 EuroQol-5+Dimension.ti,ab. (690)

26 "EQ-5D".ti,ab. (9680)

27 or/1-3 (10504)

28 or/4-15 (651442)

29 and/27-28 (10504)

30 or/16-24 (1697513)

31 and/29-30 (200)

32 or/25-26 (9957)

33 and/31-32 (2)

--------------------------------------------------------------------------------

**(C): Abdominal wall hernia**

--------------------------------------------------------------------------------

1 (inguinal or femoral or ventral or umbilical or abdominal wall).ti,ab. (335807)

2 hernia.ti,ab. (52281)

3 hernioplasty/ (9400)

4 herniorrhaphy/ (9400)

5 hernioplasty.ti,ab. (1602)

6 herniorrhaphy.ti,ab. (2372)

7 repair+or+surg*.ti,ab. (22)

8 hernia+adj+repair.ti,ab. (0)

9 (early adj3 (surg* or repair)).ti,ab. (28362)

10 trial.ti,ab. (657219)

11 RCT.ti,ab. (24987)

12 randomi#ed+controlled+trial.pt. (541163)

13 controlled+clinical+trial.pt. (94345)

14 case+control+stud$.ti,ab. (113048)

15 cross-sectional+stud$.ti,ab. (197037)

16 cohort+stud$.ti,ab. (244943)

17 retrospective+stud$.ti,ab. (179855)

18 observational+stud$.ti,ab. (126477)

19 (cost adj (utility or effectiv*)).ti,ab. (149693)

20 Economic+evaluation.ti,ab. (9983)

21 (quality of life or QoL or HRQoL).ti,ab. (312433)

22 EuroQol.af. (6571)

23 EQ-5D*.af. (9689)

24 and/1-2 (20870)

25 or/3-9 (40553)

26 or/10-20 (1972540)

27 or/21-23 (315435)

28 and/24-27 (129)

--------------------------------------------------------------------------------

**Table S6: Health-related quality of life (HRQoL) scores from the literature and sources**

| Condition | Source | Mean age* | Baseline EQ-5D-3L score | | One-year EQ-5D-3L score | |
| --- | --- | --- | --- | --- | --- | --- |
|  |  |  | **Females** | **Males** | **Females** | **Males** |
| Appendicitis | ^29^ | 32.80 | 0.751 | 0.768 | 0.967 | 0.989 |
| Diverticular disease | ^30^ | 68.00 | 0.649 | 0.666 | 0.866 | 0.889 |
| Abdominal wall hernia | ^31^ | 58.76 | 0.848 | 0.870 | 0.936 | 0.960 |

The three studies used the EuroQol- 5-Dimension (EQ-5D) in its 3-level (3L) version. *Mean age at trial start in the study.

**Table S7: Generalised Linear Models (GLMs) for quality-adjusted life years (QALYs) and costs, assessment of model fit according to root mean squared error (RMSE)**

| Family | Link | Degree | Appendicitis | Diverticular Disease | Abdominal wall hernia |
| --- | --- | --- | --- | --- | --- |
| QALYs |  |  |  |  |  |
| Binomial | Logit | 1 | [0.059] | [0.192] | [0.204] |
| Binomial | Logit | 2 | 0.059 | 0.192 | 0.204 |
| Binomial | Logit | 3 | 0.059 | 0.192 | 0.204 |
| Costs |  |  |  |  |  |
| Gaussian | Identity | 1 | 3530.330 | 8980.985 | [8975.387] |
| Inverse gaussian | Identity | 1 | 3533.875 | 8993.261 | 8988.748 |
| Gamma | Identity | 1 | 3532.422 | 8987.824 | 8983.084 |
| Gaussian | Log | 1 | 3525.947 | 8977.639 | 8976.490 |
| Inverse gaussian | Log | 1 | 3530.944 | 9002.912 | 9009.775 |
| Poisson | Log | 1 | 3526.854 | 8980.842 | 8980.611 |
| Gamma | Log | 1 | 3528.611 | 8988.305 | 8990.651 |
| Gaussian | Identity | 2 | 3530.321 | 8981.013 | 8975.399 |
| Inverse gaussian | Identity | 2 | 3533.866 | 8993.318 | 8988.820 |
| Gamma | Identity | 2 | 3532.425 | 8987.953 | 8983.125 |
| Gaussian | Log | 2 | [3525.900] | [8975.837] | 8976.386 |
| Inverse gaussian | Log | 2 | 3530.938 | 8999.703 | 9009.797 |
| Gamma | Log | 2 | 3528.617 | 8985.700 | 8990.591 |

Most appropriate GLMs for costs and QALYs were selected looking at RMSEs (in brackets). Degree refers to the polynomial order of the propensity score.

**Table S8: Most common operative procedures within emergency surgery window (panel A) and after emergency surgery window and up to one year (B)**

**(A): Within emergency surgery (ES) window**

|  | Acute appendicitis (N=268,144) | | | Diverticular disease (N=138,869) | | Abdominal wall hernia (N=106,432) | | | |
| --- | --- | --- | --- | --- | --- | --- | --- | --- | --- |
|  | | ES (N=247,506) | NES (N=20,638) | ES (N=15,772) | NES (N=123,097) | ES (N=62,559) | | | NES (N=43,873) |
| Most common high-volume operative procedures* (%) | | Emergency excision of abnormal appendix (63.0)  Unspecified other excision of appendix (16.6)  Emergency excision of abnormal appendix and drainage (9.9)  Unspecified emergency excision of appendix (3.6)  Emergency excision of normal appendix (1.3)  Other (0.1) | Interval appendicectomy (6.7)  Other specified other excision of appendix (4.2)  Planned delayed appendicectomy (1.6)  Total cholecystectomy (1.6)  Image controlled percutaneous drainage of lesion of abdominal cavity (0.4)  Other (0.1) | Rectosigmoidectomy and closure of rectal stump and exteriorisation of bowel (55.6)  Irrigation of peritoneal cavity (7.7)  Sigmoid colectomy and exteriorisation of bowel (6.6)  Anterior resection of rectum and exteriorisation of bowel (3.1)  Loop colostomy (2.3)  Other (14.2) | Fibreoptic endoscopic snare resection of lesion of colon (0.1)  Endoscopic division of adhesions of peritoneum (0.1)  Endoscopic snare resection of lesion of lower bowel using fibreoptic sigmoidoscope (0.1)  Freeing of adhesions of peritoneum (0.1)  Fibreoptic endoscopic resection of lesion of colon (0.0)  Other (0.0) | Primary repair of inguinal hernia using insert of prosthetic material (27.6)  Repair of umbilical hernia using sutures (17.9)  Repair of umbilical hernia using insert of prosthetic material (12.3)  Primary repair of femoral hernia using sutures (9.4)  Primary repair of femoral hernia using insert of prosthetic material (7.0)  Other (15.4) | | | Unspecified urethral catheterisation of bladder (0.5)  Ileectomy and anastomosis of ileum to ileum (0.3)  Unspecified excision of ileum (0.1)  Freeing of adhesions of peritoneum (0.1)  Omentectomy (0.0)  Other (0.0) |
| % with no ‘more common’ operative procedures** | 4.9 | | 84.9 | 10.6 | 99.7 | | 10.5 | 98.9 | |

Denominator is the total number of patients in the group. *’Other’ includes procedures with >1% volume in index admission appearing in ES window. **This Includes patients for whom no procedures were recorded and those who got ‘low-volume’ (<1%) procedures. NES: non-emergency surgery.

**(B): After emergency surgery window (ES) up to one year**

|  | Acute appendicitis (N=268,144) | | Diverticular disease (N=138,869) | | Abdominal wall hernia (N=106,432) | |
| --- | --- | --- | --- | --- | --- | --- |
|  | ES  (N=247,506) | NES  (N=20,638) | ES  (N=15,772) | NES  (N=123,097) | ES  (N=62,559) | NES  (N=43,873) |
| Most common high-volume operative procedures * (%) | Emergency excision of abnormal appendix (2.80)  Emergency excision of abnormal appendix (0.78)  Emergency excision of abnormal appendix (0.45)  Total cholecystectomy (0.31)  Unspecified urethral catheterisation of bladder (0.26)  Other (1.30) | Unspecified urethral catheterisation of bladder (4.33)  Emergency excision of abnormal appendix (4.27)  Unspecified other excision of appendix (2.81)  Planned delayed appendicectomy (1.88)  Planned delayed appendicectomy (0.92)  Other (3.98) | Closure of colostomy (9.41)  Rectosigmoidectomy and closure of rectal stump and exteriorisation of bowel (7.96)  Freeing of adhesions of peritoneum (1.27)  Freeing of adhesions of peritoneum (1.14)  Sigmoid colectomy and exteriorisation of bowel (1.00)  Other (7.23) | Fibreoptic endoscopic snare resection of lesion of colon (4.00)  Fibreoptic endoscopic resection of lesion of colon (1.74)  Rectosigmoidectomy and closure of rectal stump and exteriorisation of bowel (1.21)  Endoscopic snare resection of lesion of lower bowel using fibreoptic sigmoidoscope (1.04)  Anterior resection of rectum and anastomosis of colon to rectum using staples (0.60)  Other (3.72) | Primary repair of inguinal hernia using insert of prosthetic material (3.83)  Repair of umbilical hernia using sutures (1.70)  Unspecified urethral catheterisation of bladder (1.52)  Repair of umbilical hernia using insert of prosthetic material (1.14)  Repair of recurrent inguinal hernia using insert of prosthetic material (0.69)  Other (3.01) | Primary repair of inguinal hernia using insert of prosthetic material (26.9)  Repair of umbilical hernia using insert of prosthetic material (6.17)  Repair of umbilical hernia using sutures (5.56)  Repair of recurrent inguinal hernia using insert of prosthetic material (2.86)  Unspecified urethral catheterisation of bladder (1.49)  Other (6.30) |
| % with no ‘more common’ operative procedures** | 94.1 | 81.8 | 72.0 | 87.7 | 88.1 | 50.7 |

Denominator is the total number of patients in the group. *’Other’ includes procedures with >1% volume in index admission appearing after the ES window. **This Includes patients for whom no procedures were recorded and those who got ‘low-volume’ (<1%) procedures. NES: non-emergency surgery.

**Table S9: Summary of sensitivity analyses (SA) results. Overall Incremental Net monetary Benefit (INB) of emergency surgery (ES) vs non-emergency surgery (NES) strategies**

| Analysis | Description | Acute appendicitis (N=268,144) | Diverticular disease (N=138,869) | Abdominal wall hernia (N=106,432) |
| --- | --- | --- | --- | --- |
| Base case | See Main document | -86.2 (-1,163, 991) | 2,664 (-4,298, 9,626) | -119 (-1,282, 1,043) |
| SA1 | Considered alternative measures of hospital quality derived from the *National Emergency Laparotomy Audit (NELA)* reports from 2016-2018 (see Section 5) | 408 (-787, 1,605) | 5,823 (1,029, 10,616) | 125 (-1,027, 1,276) |
| SA2 | Considered a 10% decrease in all unit costs in total cost calculation (see Section 1) | -96.9 (-1,078, 884) | 2,491 (-3,673, 8,655) | -30.4 (-964, 903) |
| SA3 | Considered a 10% increase in all unit costs in total cost calculation (see Section 1) | -75.2 (-1,257, 1,107) | 2,836 (-4,356, 10,028) | -208 (-1,254, 837) |
| SA4 | Used linear interpolation between baseline and one-year HRQOL endpoints for calculating QALYs (see Section 2) | -202 (-1,514, 1,110) | 2796.432 (-2,796, 8,389) | -125 (-1,216, 967) |
| SA5 | Evaluated costs and effects of ES and NES over a five-year time horizon (see Section | -3,786 (-9,113, 1,541) | -1,502 (-27,066, 24,062) | 700 (-6,812, 8,212) |

INB of ES in the sensitivity analyses was estimated using Local Instrumental Variable methods. 95% confidence interval in parentheses. ES: emergency surgery, HRQoL: health-related quality of life, NES: non-emergency surgery, QALYs: quality-adjusted life years.

**Supplemental figures**

**Figure S1: Variation in tendency to operate (TTO) across 175 NHS hospitals in the one year prior to emergency admissions that meet the inclusion criteria for appendicitis (panel A), diverticular disease (panel B) and abdominal wall hernia (panel C)**

**(A): Appendicitis (C): Abdominal wall hernia**

**
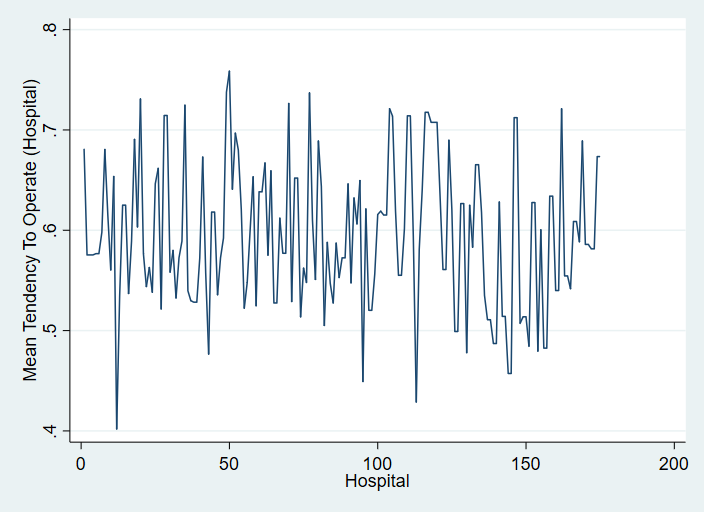
**
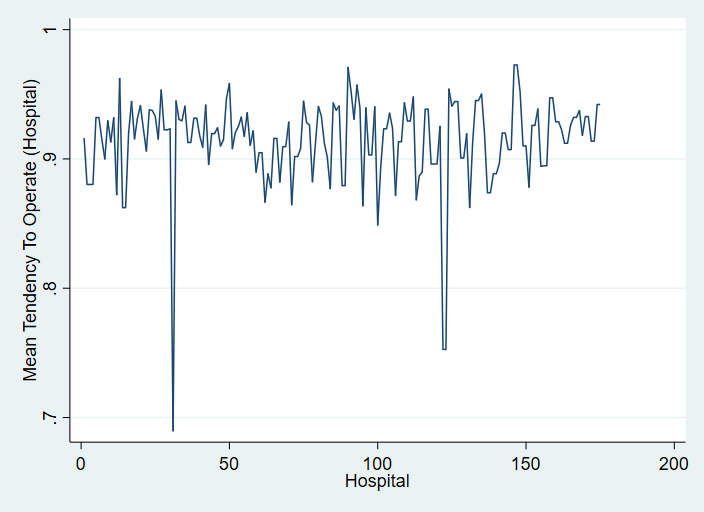


**(B): Diverticular disease**

***
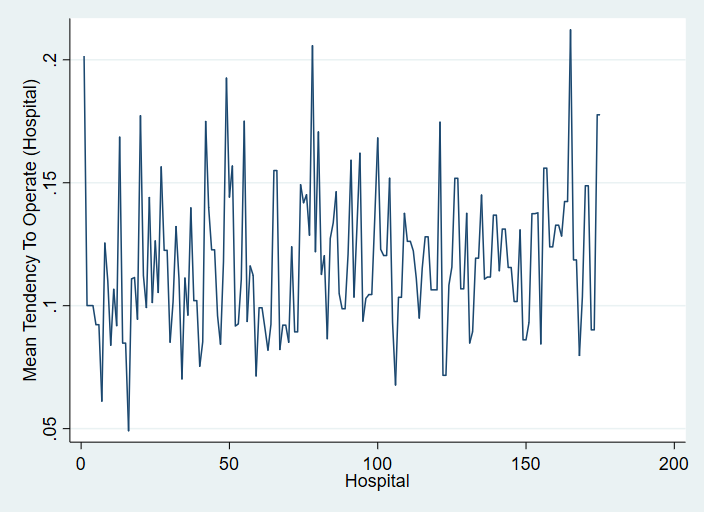
***

**Figure S2: Kaplan-Meier estimates for time to one-year death for appendicitis (panel A), diverticular disease (B), abdominal wall hernia (C)**

**(A): Appendicitis (C): Abdominal wall hernia**


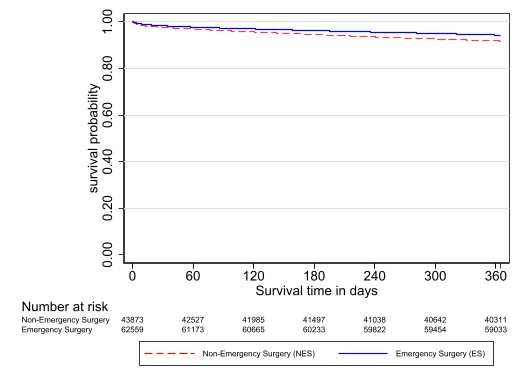

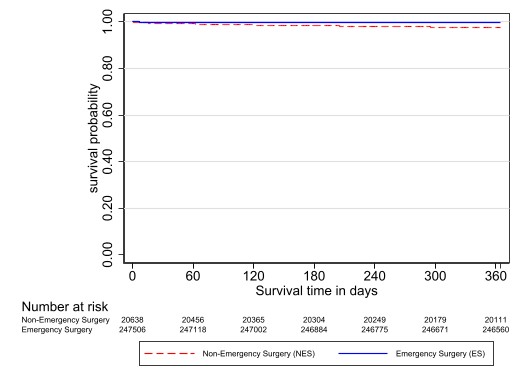


**(B): Diverticular disease**


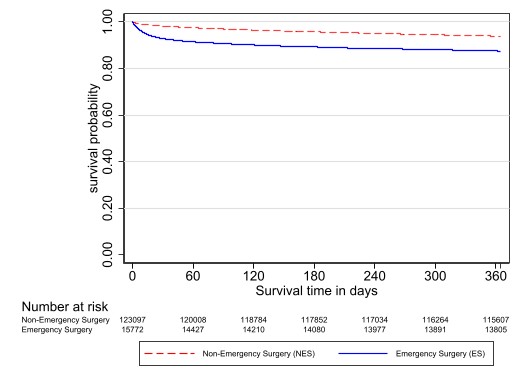


**Figure S3: Forest plots of estimated incremental costs and Quality-adjusted Life Years (QALYs) from the Local Instrumental Variables (LIV) approach**

**(A): Appendicitis**


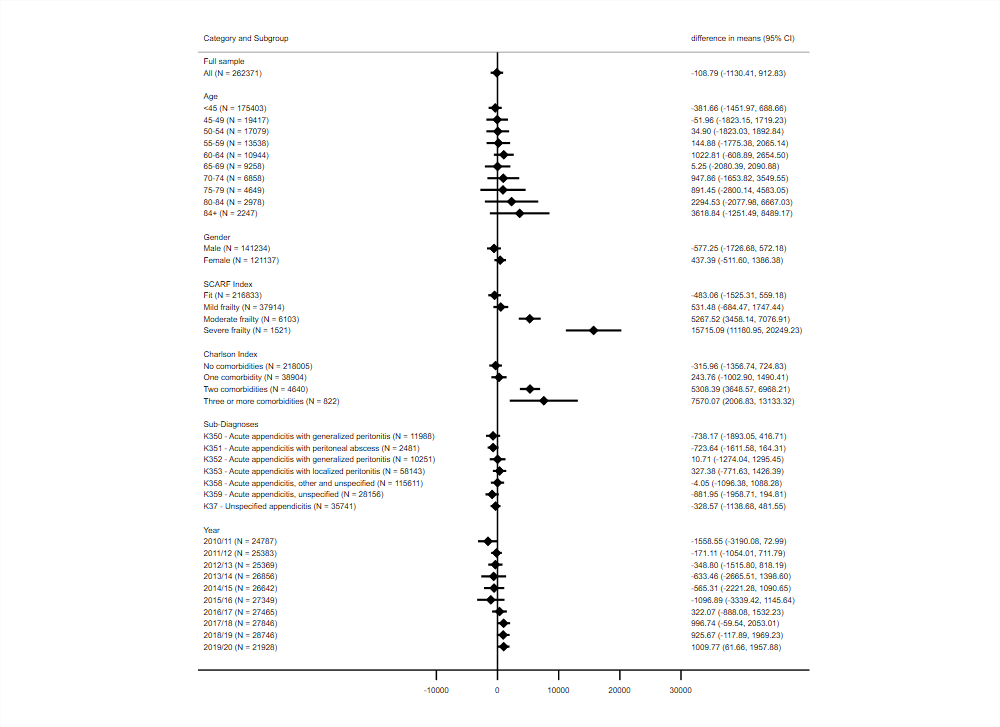

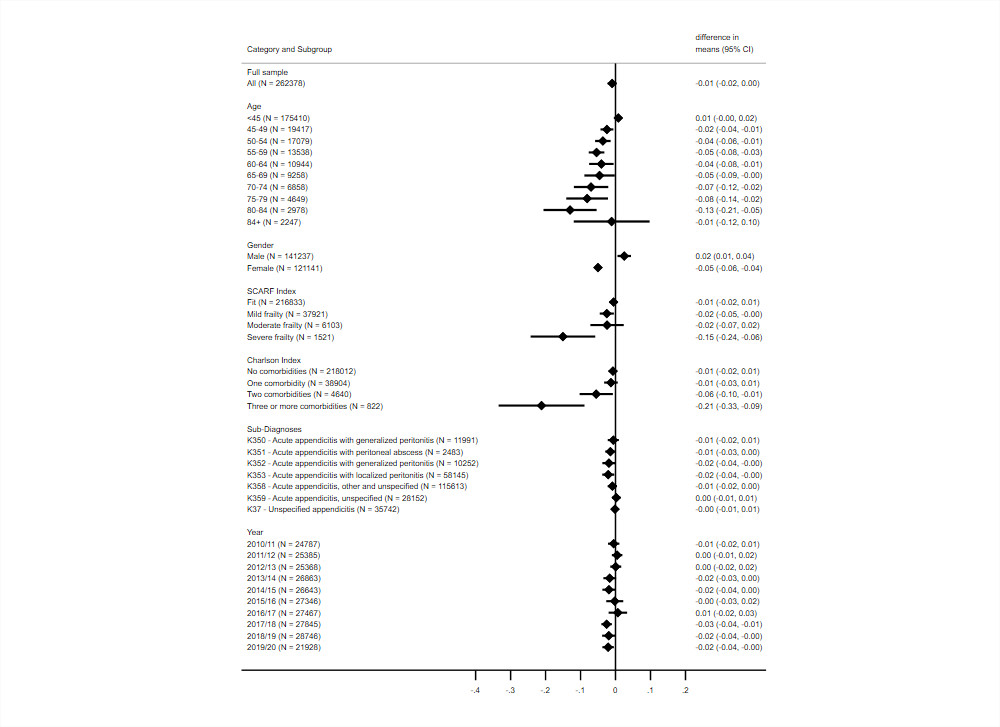


**(B): Diverticular disease**


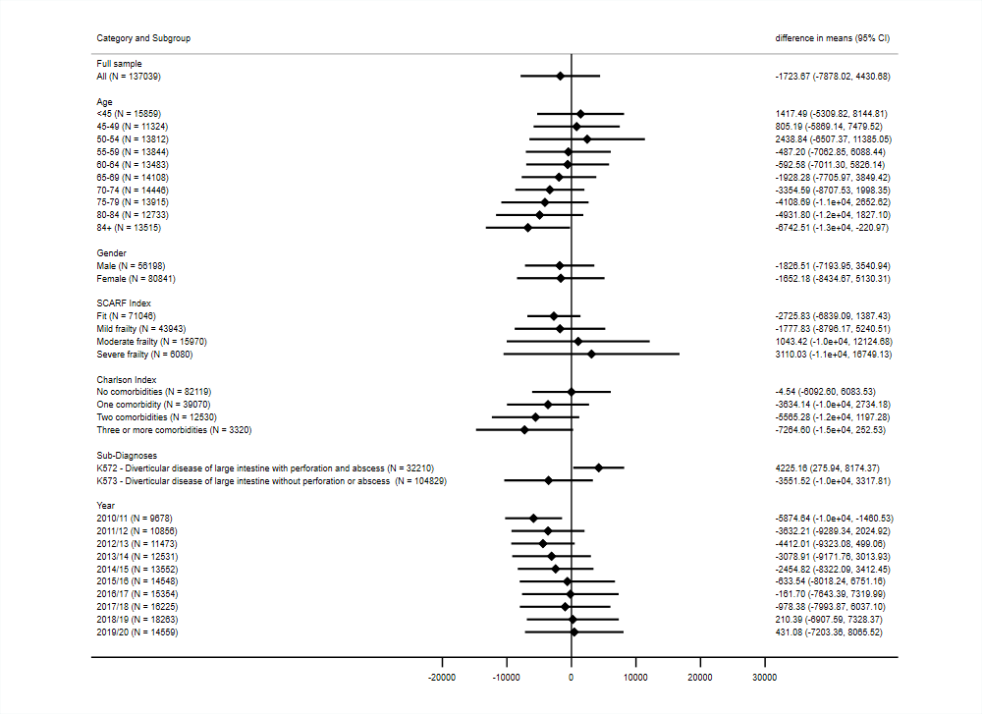

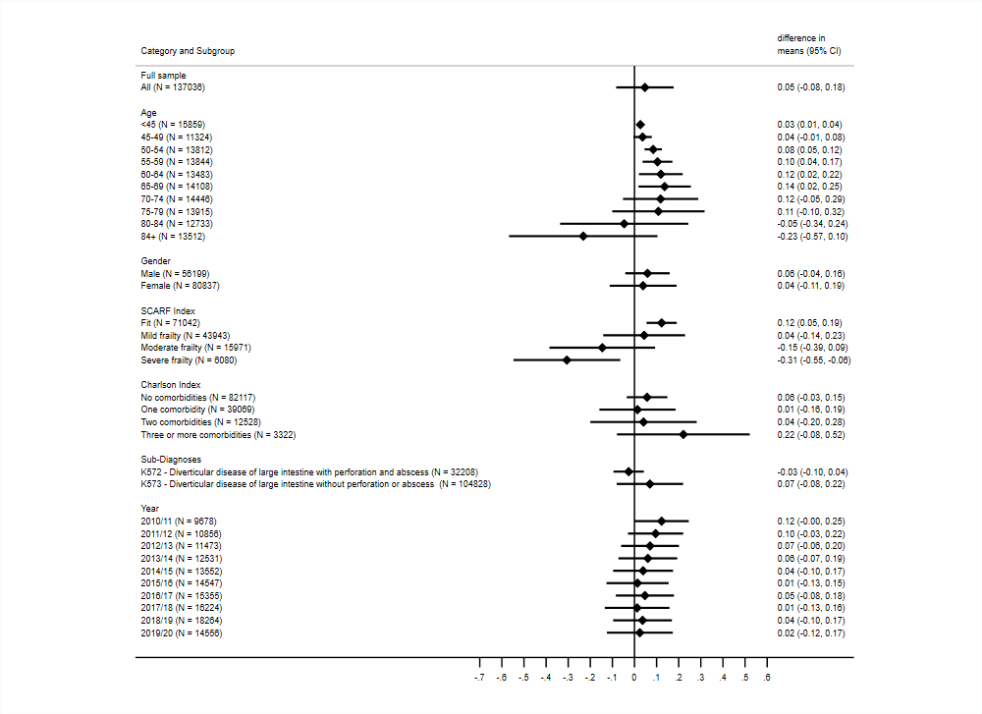


**(C): Abdominal wall hernia**


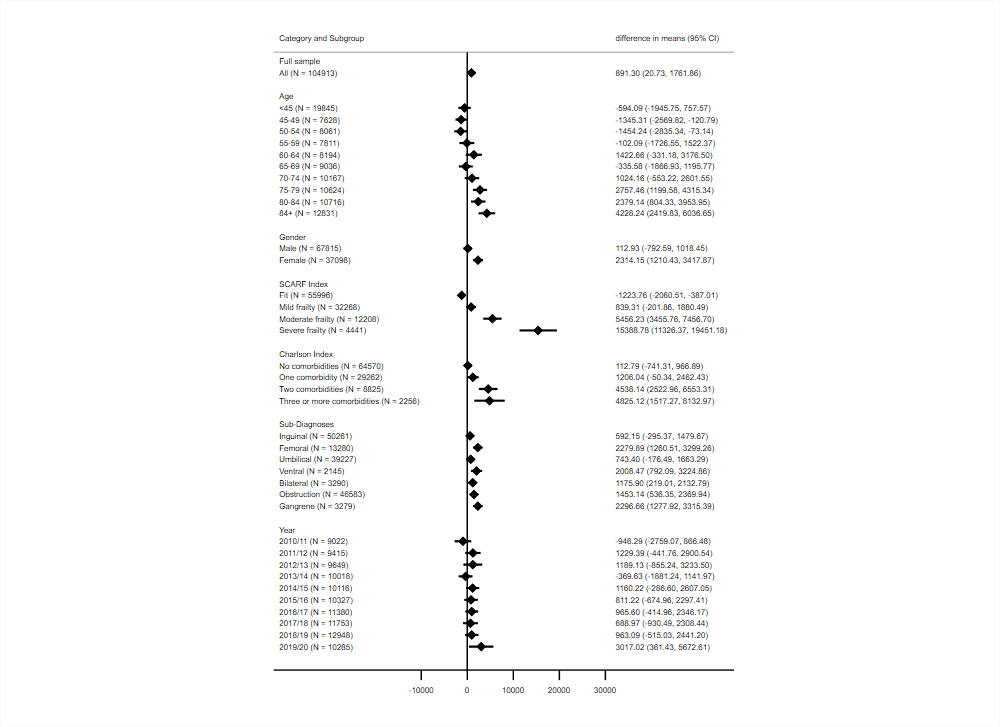

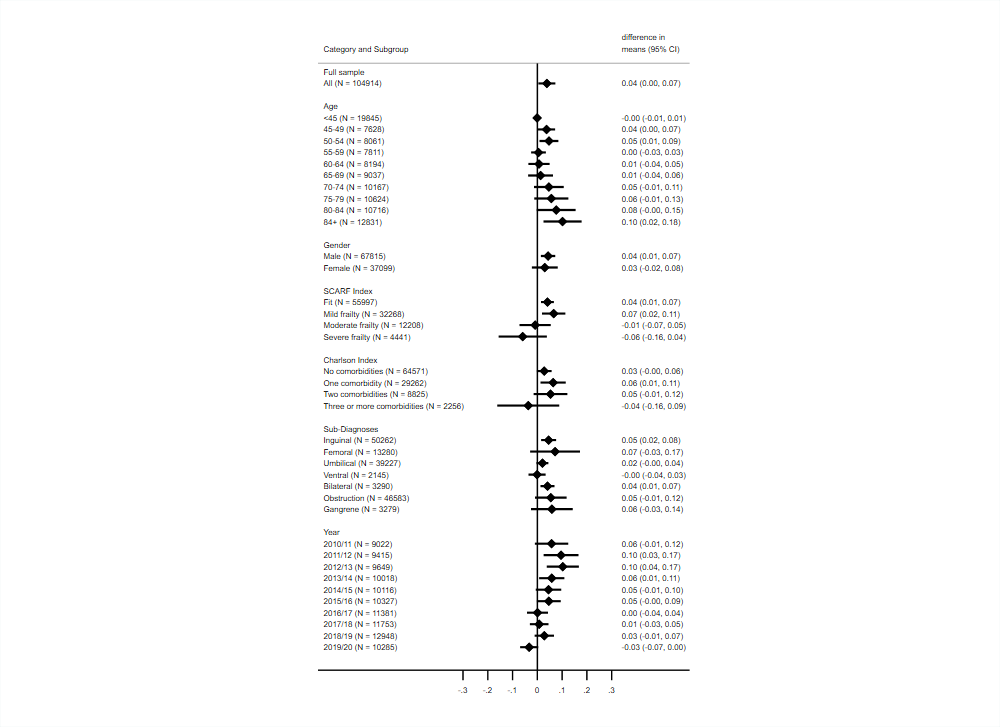


**Figure S4: Health-related Quality of Life (HRQoL) trajectory following initial (index) emergency admission and emergency readmission for the base case, which assumes HRQoL reaches follow-up levels following hospital discharge (panel A), and linear interpolation (B, sensitivity analysis 4)**


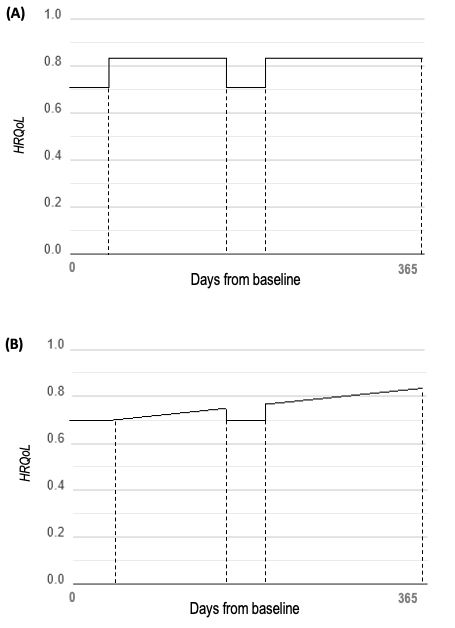


(A) Immediate interpolation. Baseline HRQoL is assumed to apply constantly for the duration of the index admission and any emergency readmission. Following the index admission, the HRQoL is assumed to apply constantly for the duration of the period before the final (one-year) endpoint, which is accrued immediately after discharge. (B) Linear interpolation. Baseline HRQoL is assumed to apply constantly for the duration of the index admission and any emergency readmission. HRQoL between the endpoints is assumed to increase linearly.

**(B)**

Days from baseline

Days from baseline

**0**

**0**

**365**

**365**

*HRQoL*

*HRQoL*

1. The same 1% rule was used for identifying the most common diagnostic procedures that were costed. [↑](#footnote-ref-1)
